# *Interactive* Multiple Object Tracking (iMOT)

**DOI:** 10.1371/journal.pone.0086974

**Published:** 2014-02-03

**Authors:** Ian M. Thornton, Heinrich H. Bülthoff, Todd S. Horowitz, Aksel Rynning, Seong-Whan Lee

**Affiliations:** 1 Department of Cognitive Science, University of Malta, Msida, Malta; 2 Psychology Department, Swansea University, Swansea, United Kingdom; 3 Max Planck Institute for Biological Cybernetics, Tübingen, Germany; 4 Department of Brain and Cognitive Engineering, Korea University, Seoul, Republic of Korea; 5 Visual Attention Laboratory, Brigham and Women's Hospital, Boston, Massachusetts, United States of America; 6 Department of Ophthalmology, Harvard Medical School, Boston, Massachusetts, United States of America; University of California, Davis, United States of America

## Abstract

We introduce a new task for exploring the relationship between action and attention. In this interactive multiple object tracking (iMOT) task, implemented as an iPad app, participants were presented with a display of multiple, visually identical disks which moved independently. The task was to prevent any collisions during a fixed duration. Participants could perturb object trajectories via the touchscreen. In Experiment 1, we used a staircase procedure to measure the ability to control moving objects. Object speed was set to 1°/s. On average participants could control 8.4 items without collision. Individual control strategies were quite variable, but did not predict overall performance. In Experiment 2, we compared iMOT with standard MOT performance using identical displays. Object speed was set to 2°/s. Participants could reliably control more objects (M = 6.6) than they could track (M = 4.0), but performance in the two tasks was positively correlated. In Experiment 3, we used a dual-task design. Compared to single-task baseline, iMOT performance decreased and MOT performance increased when the two tasks had to be completed together. Overall, these findings suggest: 1) There is a clear limit to the number of items that can be simultaneously controlled, for a given speed and display density; 2) participants can control more items than they can track; 3) task-relevant action appears not to disrupt MOT performance in the current experimental context.

## Introduction

Psychologists have long been interested in the extent to which we can divide attention [1]–[3]. Across a wide range of experimental paradigms, the general finding has been that while it is clearly possible to allocate attention to more than one object or event, such division almost always results in performance costs, particularly when overall processing demands are high [4]–[6]. Outside of the laboratory, the requirement to divide attention during daily life appears to be ever increasing. The proliferation of mobile technology, for example, often leads to situations where a private information stream, such as a text or e-mail message, is being processed in parallel with a more public activity, such as walking in a crowded street, watching TV with friends, or even holding a face-to-face conversation. One critical situation where the limits of dividing attention become highly relevant is driving. David Strayer and colleagues, for example, have demonstrated that almost any interaction with a mobile device while driving a car can impair vehicle control and situational awareness to a level where lives are put at risk [7]–[8].

The multiple object tracking paradigm (MOT, [9]) has proven to be a very useful laboratory tool for exploring the limits of dividing attention in complex, dynamic contexts (for a review see [10]). In a typical display, observers are shown a fixed number of identical objects. Half of the objects are identified as targets, by briefly highlighting or blinking them. With the highlighting removed, the display is set in motion, with all of the (now identical) objects following random, independent trajectories. At the end of a variable tracking period, the motion stops and the observer is probed for the identity of the target set. The dependent measure is the inferred proportion of targets correctly tracked [11].

The MOT task has proven popular, at least in part, because the displays appear to capture some of the complexity that we might encounter in our day-to-day environment. With this in mind, there are two main findings of particular interest that have emerged. The first is simply that observers are actually able to do the task. That is, MOT is a very powerful demonstration that attention can be divided and controlled across multiple objects for sustained periods of time, despite the motion of the objects across the display. The second major finding is that such attentive tracking is limited to 3–5 items. While observers have no trouble perceiving the motion of dozens or even hundreds of objects, they can only track a handful (cf. [12]). Several explanations have been proposed for this limit, including a fixed set of virtual pointers [13]–[14], flexible attentional resources [15], and limitations in oscillation phase space [16].

The purpose of the current work was to explore how *action* might influence such limits. The relationship between action and attention has been well established. Indeed some theorists have even suggested that it is the limited capacity to act that determines attentional resources [17]–[19]. Planning an action clearly has important consequences for the deployment of attention. For example, it can facilitate processing at intended target locations [20]–[21] and can modulate the salience of object features [22]–[23] and object groupings [24]–[25].

The majority of these action-attention findings relate to the selection of single targets (i.e. focused attention). How does the need to act influence the deployment and control of divided attention? Here, we introduce a new task aimed at answering this question by exploring performance limits when individuals must *interact* with multiple objects in addition to simply tracking them. Our aim was both to explore the influence of action during divided attention tasks and to extend MOT to more fully capture the active dimension of day-to-day life that has thus far been ignored in laboratory studies.

### interactive Multiple Object Tracking (iMOT)

**Figure 1:**
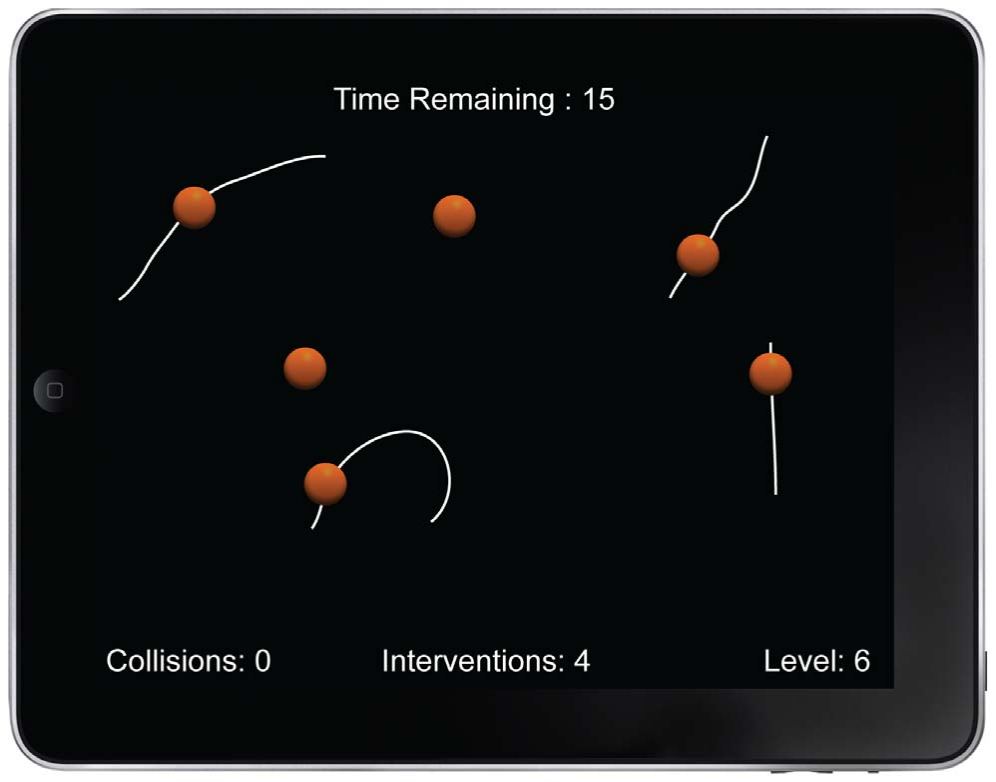
A typical iMOT display for a trial at Level 6 in Experiment 1. The spheres move at random unless a path is drawn by touching and dragging away with a linear or curved movement. The white path remains visible until the path has been traversed; the object then resumes random motion. The task is to avoid collisions. The timer at the top of the screen counts down from 30 seconds to zero, and the fields along the bottom of the screen provide information about the current trial status.

The task we introduce in the current paper is illustrated in Figure 1. Similar to standard MOT tasks, it consists of a visual display in which multiple identical objects move at random. However, instead of passively tracking the targets, the goal of iMOT is to actively prevent objects from colliding. In designing this task, we wanted to exploit what we see as an interesting difference between laboratory MOT and the real world tasks that seem most directly analogous to it.

When we track objects outside the laboratory, there may be some situations in which we simply want to passively follow objects of interest while ignoring distractors, such as when watching sporting events. However, in many other situations, in addition to tracking, we must also interact with and/or control elements of our environment. To return to an earlier example, when driving in heavy traffic or approaching a busy junction, we need to track and predict the behavior of other vehicles as well as control our own position in space. In CCTV monitoring stations and air traffic control rooms, operators need to both attend to and control multiple channels. For CCTV operators, tracking a group of individuals through an environment requires selection and control of multiple cameras [26]. For air traffic control (ACT) tasks, all designated planes are relevant objects and, particularly during the approach/departure phase, specific actions are required to achieve collision-free allocation of appropriate airspace/runways [27].

The design of our experiments was directly inspired by mobile games such as Flight Control (Firemint Pty Ltd) and Harbor Master (Imangi Studios, LLC) that mimic aspects of the ACT task. In these games, which are typically implemented on touchscreen devices such as the iPad (Apple, Inc.), the players try to keep planes and ships from colliding, while directing them to the appropriate runways or harbors.

In designing the iMOT task, then, we moved away from asking participants to localize the target set or discriminate between targets and distractors. Instead, all objects become targets and the goal is to prevent any object from coming into contact or colliding with any other object. Participants are given control over the trajectories of the objects using the standard touch interface implemented on most mobile devices. Touching and dragging away from an object with a finger either creates a visible path for the object to follow (Experiment 1) or nudges the object in the appropriate direction (Experiments 2 & 3). We note that although the initial iMOT experiments have been carried out on an iPad, the paradigm can easily be implemented on any device with similar displays and touch-response capabilities.

### iMOT Task Demands

To perform well in this task participants need to both monitor for impending collisions and to plan and execute motor interventions aimed at keeping specific objects apart. Collision detection might involve actively tracking objects, in the manner of MOT. Alternatively, there might be a more passive collision-detection system. Collision detection might be based on simple proximity, so that a signal is generated if two items get too close to one another, or a more sophisticated algorithm might take into account the speed and trajectory of items to predict potential collisions.

Collision detection is also involved in MOT, of course. Work by Zelinsky and Todor [28] has shown that the visual system responds proactively to potential collisions, shifting gaze to the relevant location in advance in order to help disambiguate potential collisions (Zelinksy and Todor call this behavior “rescue saccades”). However, little or no work has been done on determining “how” collisions are detected in multiple object displays. While there is some evidence that MOT involves prediction [29]–[31], at least in predictable displays [32], we do not know if this extends to collision detection.

The iMOT task differs from MOT in that the participant can actively respond to potential collisions. Consider two possible strategies that might be adopted. A participant with a *reactive* approach might wait until an collision seemed imminent before taking steps to avoid it. This strategy is similar to the “rescue saccades” described in MOT by Zelinsky and Todor [28]. A participant with a *proactive* approach, on the other hand, might try to continually modify the position of objects on the screen to maximize the distance between them, thus reducing the likelihood of collisions. As the two approaches would likely give rise to quite different touch behavior, examining intervention style should be able to shed light on which approach is more prevalent.

Although the overall goals of iMOT and MOT are quite different – collision avoidance versus target identification – in terms of task demands, collision management may be a shared requirement. That is, while the goal of iMOT is to avoid collisions, in MOT it may be useful to have a strategy to minimize the impact of collisions on tracking. Thus, the two tasks may rely on a common collision detection mechanism (active or passive), or they may not. In the current paper, we attempt to directly compare iMOT and MOT performance in the same participants as a way to initially assess whether tracking and collision avoidance involve similar cognitive processes. We should note that in making such comparisons we intend to focus purely on performance measures. That is, we do not assume, *a priori*, that behavior is limited by a fixed set of task-specific mechanism [13]–[14] rather than being determined via the flexible allocation of central attentional resources [15].

As already mentioned above, one of the main aims of this paper is to explore the influence of action when attention needs to be divided. To successfully avoid collisions during iMOT, actions must be planned and effectively executed with respect to a single object at a time. Understanding whether such focused action has consequences for the ability to monitor other parts of the display should become clear by examining iMOT performance. Furthermore, by directly comparing iMOT to MOT performance in the same participants, we hope to shed light on whether these two components – action and attention – operate independently or rely on overlapping cognitive resources.

### Experimental Overview

Experiment 1 was intended as “proof of concept”, to demonstrate that observers could in fact successfully perform the iMOT task. We chose a speed of object movement (1°/s) that was relatively sedate, taking into account the need for physical control, and used a staircase procedure to obtain individual thresholds and control distributions. We were interested in both the absolute number of items that could be controlled and the variability of this figure across participants. One prominent feature of MOT is the finding that, in the majority of displays, estimates of tracking capacity show little individual variability around the oft-cited limit of 4–5 items [10]. Factors that are known to modulate group mean estimates in MOT tasks, such as speed of motion [15], [33], set size and display density [34]–[37], are also discussed in Experiment 1.

In Experiment 2, we directly compared MOT and iMOT performance. Using identical displays and motion parameters, we obtained estimates of both MOT tracking performance and iMOT control performance in the same individuals. The goal was to establish the relative demands of the two tasks and to assess whether performance on MOT and iMOT appeared to be drawing on similar resources.

In Experiment 3, we used a dual-task approach to examine whether MOT and iMOT could be performed simultaneously. In single-task displays, participants either tracked or controlled four target objects. Difficulty was manipulated by changing the distractor set-size. In the critical dual-task condition, the same four objects had to be both tracked for later identification and controlled to avoid collisions. Under these conditions, we were interested in establishing how resources would be balanced between the two tasks. If MOT and iMOT relied on completely separate resources, then dual-task performance should be comparable to the single-task baselines. The presence of a dual-task deficit would indicate some overlap in processing resources.

## Experiment 1

The purpose of Experiment 1 was to demonstrate that participants could successfully perform the iMOT task. We compared two groups of participants. The first consisted of young adults from Korea University in Seoul. The second were young adults from Swansea University in the UK. The motivation for including this cross-cultural variable, aside from the availability of separate pools of participants, was to probe for possible differences in cognitive style. Previous research has suggested that there may be fundamental differences between East Asian and Western participants, with the former attending more to background context, and the latter to figural elements [38]–[39]. Such differences could potential impact performance in the current task.

Previous research has also suggested that there may be sex differences in spatial selective attention [40]–[42], specifically in the context of multiple object tracking [43]. Therefore, we ensured that each group consisted of an equal number of male and female participants, and we included sex as a factor for exploratory purposes in all three experiments.

### Method

#### Participants

A total of 24 participants took part in this study on a voluntary basis. A group of 12 younger adults (six female and six male) aged between 18–26 years (M = 24.1, SD = 2.5) were recruited directly from members of the Brain Engineering Department at Korea University. A further group of 12 younger adults (six female and six male), aged between 19–33 years (M = 23.2, SD = 4.3), were recruited from the Psychology Department at Swansea University. All participants were asked to assess their familiarity with game-like tasks on mobile devices on a scale from 1 (no experience) to 5 (expert player). There were no differences between the Korean group (M = 3.2, SD = 1.2) and the Swansea group (M = 3.3, SD = 1.0), *t*<1, n.s. All participants gave written informed consent, and the methods and procedures conformed to the ethical guidelines set out by the Declaration of Helsinki for testing human participants. All aspects of the procedure was reviewed and approved by the Ethics Committee at Swansea University.

#### Equipment

All experiments reported here used a first generation iPad with a screen dimension of 20×15 cm and a resolution of 1024×768 pixels. In this and all subsequent experiments, participants were instructed to hold the iPad in a standard posture: the participant cradled the iPad (in landscape orientation) in their left arm, with the fingers of their left hand grasping the furthest edge of the device. They were told to interact with the objects using the index finger of their right hand. While the viewing distance was not fixed, we estimate that it averaged approximately 50 cm from screen surface to eyes. For this reason, we report stimulus characteristics both in terms of approximate degrees of visual angle (°) and pixels. Text was set to run from left-to-right.

Experiments were run in a quiet environment under low lighting conditions with no overhead lights, in order to minimize screen glare.

#### Stimuli and Task

The iMOT task was introduced to participants as a simple game in which the goal was to prevent moving objects from colliding with each other. All participants began with a display containing six objects. If they successfully controlled these objects without collision for 30 s, an additional object would be added on the subsequent trial. Any collision between two objects ended a trial. After a collision, the number of objects would be reduced but would never go below the initial level of six items. Performance was assessed over a total of 30 trials per participant. From a player's perspective, success in the game involved achieving and maintaining the highest level (i.e., greatest number of objects) possible.

Objects were identical orange spheres with a diameter of 52 pixels (1.2°). The objects were shaded to appear lit from above. This was done to enhance the impression of 3D and help segment them from the uniform black background. At the start of each trial, the objects were distributed at equal distances around the circumference of an invisible circle centered on the iPad display. The radius of this circle was 160 pixels (3.1°). The position of spheres around the circle was determined by choosing a random starting angle for the first object and then distributing each subsequent object by adding an equidistant angular step of (360/Set Size) °.The objects were stationary for the first two seconds of the trial, and then began to expand outwards in a straight line, following an angular trajectory equivalent to their position around the circle. In the absence of participant input, each object followed this path for 200 pixels, when a new straight line path would be selected at random. Directions were randomly sampled from the full 360° in 1° increments and the path length varied between 200 pixels (3.9°) and 300 pixels (5.9°). At all times, objects moved at a constant speed of approximately 1°/s.

The participant's task was to keep the objects separated by perturbing their trajectories via the touchscreen interface. Touching and dragging away from an object gave rise to a visible white path that the object would follow. In this experiment, the length and complexity of the path was not restricted. When the object reached the end of a user defined path, it reverted to following random linear paths, as described above. Note, that user input was allowed immediately at the start of the trial, that is, within the first two seconds. In these circumstances, the user defined path would override the default linear expansion for the touched object.

In line with the idea of “game-play”, four information fields were visible to the participants during the entire trial. At the top of the screen was a time counter that reduced from 30 s to 0 s. In the bottom left corner was a collision counter and in bottom right an indication of the current number of objects in the display. At the bottom of the screen in the center was an indication of the number of touches or interventions made during the current trial.

#### Procedure

Participants were run in individual sessions. Each session began with a brief questionnaire aimed at establishing educational and work experience, gaming habits and familiarity with mobile devices. Questions were a mixture of open-ended items and rating scales designed to quantify relevant experience. This lasted approximately 5 minutes. Participants were then familiarized with the iPad and the basic display and control components of the task. They were allowed to practice with the application until they felt comfortable. This familiarization phase typically lasted less than 5 minutes, with participants completing two or three practice trials. The main experimental session then began in which participants completed a block of 30 trials, each trial lasting 30 s. At the end of each trial, a self-paced pause was allowed. Participants could wait as long as long as they liked until pressing a “Continue” button. In practice, few of these paused lasted more than 10 seconds, with the entire block being completed within 20 minutes.

#### Analysis

The main dependent measure in Experiment 1 was the number of objects that could be successfully controlled for 30 seconds without collision. Our analyses thus focused on the distribution of collision-free trials as a function of set size. As well as reporting the mean of these distributions, in this and all subsequent experiments, we also extracted a full range of parameters (i.e., variability, skewness, kurtosis, maximum) that might help characterize performance. These will be reported in the accompanying tables, but analysis will focus on the central tendency and the maximum level achieved. In Experiment 1, these dependent variables were analyzed using a 2 (Group: KU vs. SU) ×2 (Sex) ANOVA.

We also looked at how often participants touched the objects to change their trajectories, which we termed “interventions”. We calculated the average number of interventions per collision-free trial, and fitted a line to the intervention x set size function of each participant. Both the slope of this function, and the baseline interventions with a set size of 6 items were examined. Average interventions were analyzed using a 2 (Group: KU vs. SU) ×2 (Sex) ×5 (Set Size) ANOVA, while slope and baseline measures used a 2 (Group: KU vs. SU) ×2 (Sex) ANOVA.

Finally, we looked to see whether intervention strategy had any impact on overall performance. To do this we used multiple regression to explore whether the number of items controlled could be predicted from the slope and baseline interaction measures.

### Results

**Figure 2:**
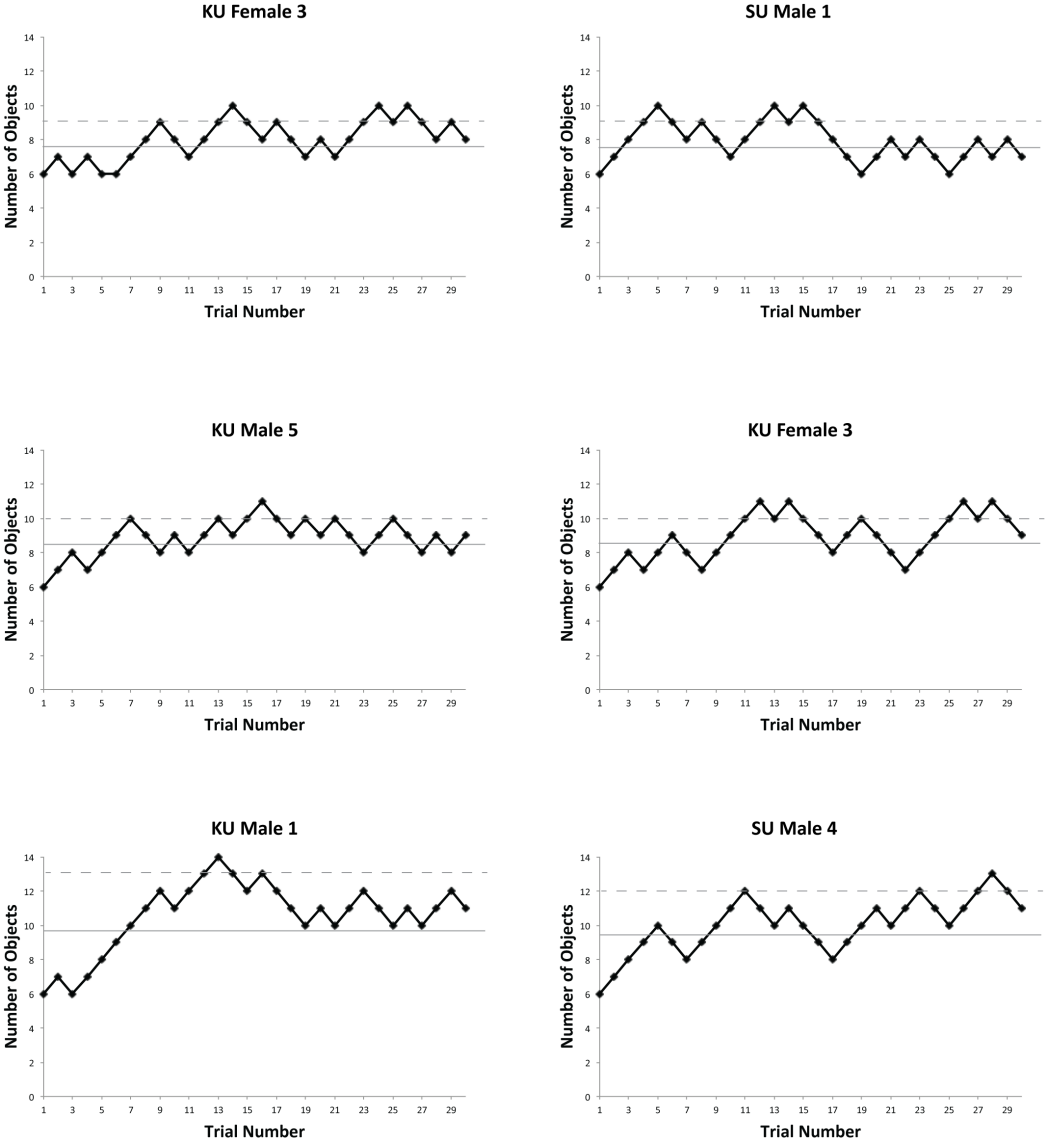
Example staircase data in Experiment 1. The left hand column shows data from three Korea University (KU) participants, the right column three Swansea University (SU) participants. The Y-axis indicated the number of items in the current trial, and each data point represents one of the 30 trials in a session. A collision-free trial always results in an increase in set size while any collision results in a decrease, except that set size was not allowed to drop below six items. The solid line shows the mean level achieved by the participant and the dotted line the maximum level. See text for more details.

Figure 2 shows examples of individual staircase sessions for six participants, three from KU in the left hand column, three from SU in the right hand column. In each panel, the solid line indicates the mean and the dashed line the maximum number of items controlled for that individual. The panels are labeled so that data from the corresponding participant can be found in Tables 1 and 2.

**Table 1:** Korea University participants from Experiment 1: Individual Parameter Estimates for Distributions of Collision-free Trials.

	Female						Male					
Part.	Count	Mean	Var	Skew	Kurt	Max	Count	Mean	Var	Skew	Kurt	Max
1	17	9.59	2.26	−0.94	1.05	12	18	9.89	3.99	−0.78	−0.02	13
2	16	7.63	1.05	0.46	0.83	10	17	8.41	1.26	−0.35	−0.11	10
3	16	7.56	1.06	−0.19	−0.95	9	16	7.94	1.00	0.14	0.22	10
4	16	7.88	0.65	−0.63	0.75	9	18	8.67	1.65	−0.23	−0.23	11
5	16	8.56	1.46	−0.55	−0.32	10	17	8.29	0.97	−0.68	0.55	10
6	17	7.88	1.11	−0.10	0.16	10	17	8.47	1.51	0.08	0.28	11
Mean	16.33	8.18	1.27	−0.33	0.26	10.00	17.17	8.61	1.73	−0.30	0.12	10.83
SE	0.23	0.34	0.25	0.22	0.35	0.49	0.34	0.30	0.51	0.17	0.13	0.52

**Table 2:** Swansea University participants from Experiment 1: Individual Parameter Estimates for Distributions of Collision-free Trials.

	Female						Male					
Part.	Count	Mean	Var	Skew	Kurt	Max	Count	Mean	Var	Skew	Kurt	Max
1	17	7.88	1.99	0.23	−1.21	10	16	7.38	1.05	0.39	−0.80	9
2	17	8.35	1.62	−0.14	−1.07	10	17	8.71	1.72	−0.32	−0.26	11
3	16	7.75	1.27	0.24	−0.40	10	16	9.19	2.03	−0.69	0.27	11
4	16	7.75	2.07	0.19	−1.38	10	18	9.44	2.50	−0.54	−0.17	12
5	18	9.17	2.50	−0.31	−0.39	12	17	9.00	1.75	−0.55	0.39	11
6	17	7.88	1.11	−0.47	−0.93	9	17	8.06	1.06	−0.13	−0.32	10
Mean	16.83	8.13	1.76	−0.04	−0.90	10.17	16.83	8.63	1.68	−0.31	−0.15	10.67
SE	0.34	0.25	0.23	0.14	0.19	0.44	0.34	0.35	0.25	0.18	0.19	0.46

In the upper row are two participants whose performance fluctuated around the lower end of the range. In the first example (KU Female 3), performance initially stays close to the starting level of 6 items, but gradually rises to fluctuate between 7 and 9 items, never exceeding this maximum level. The second example (SU Male 1) also has a maximum of 9 items, but here there is an initial rise and fall, which is repeated before performance stabilizes around 7 items in the latter half of the session. The second row of examples shows participants who were able to successfully control at least 10 items without collision. For KU Male 5, this only occurs once (at trial 16), and performance seems to stabilize for this participant at 9 items. SU Female 2 is able to control 10 items on 4 occasions, but their overall performance shows a more periodic increase and decrease. The final two examples show those participants with the highest sustained performance from the two sites.

**Figure 3:**
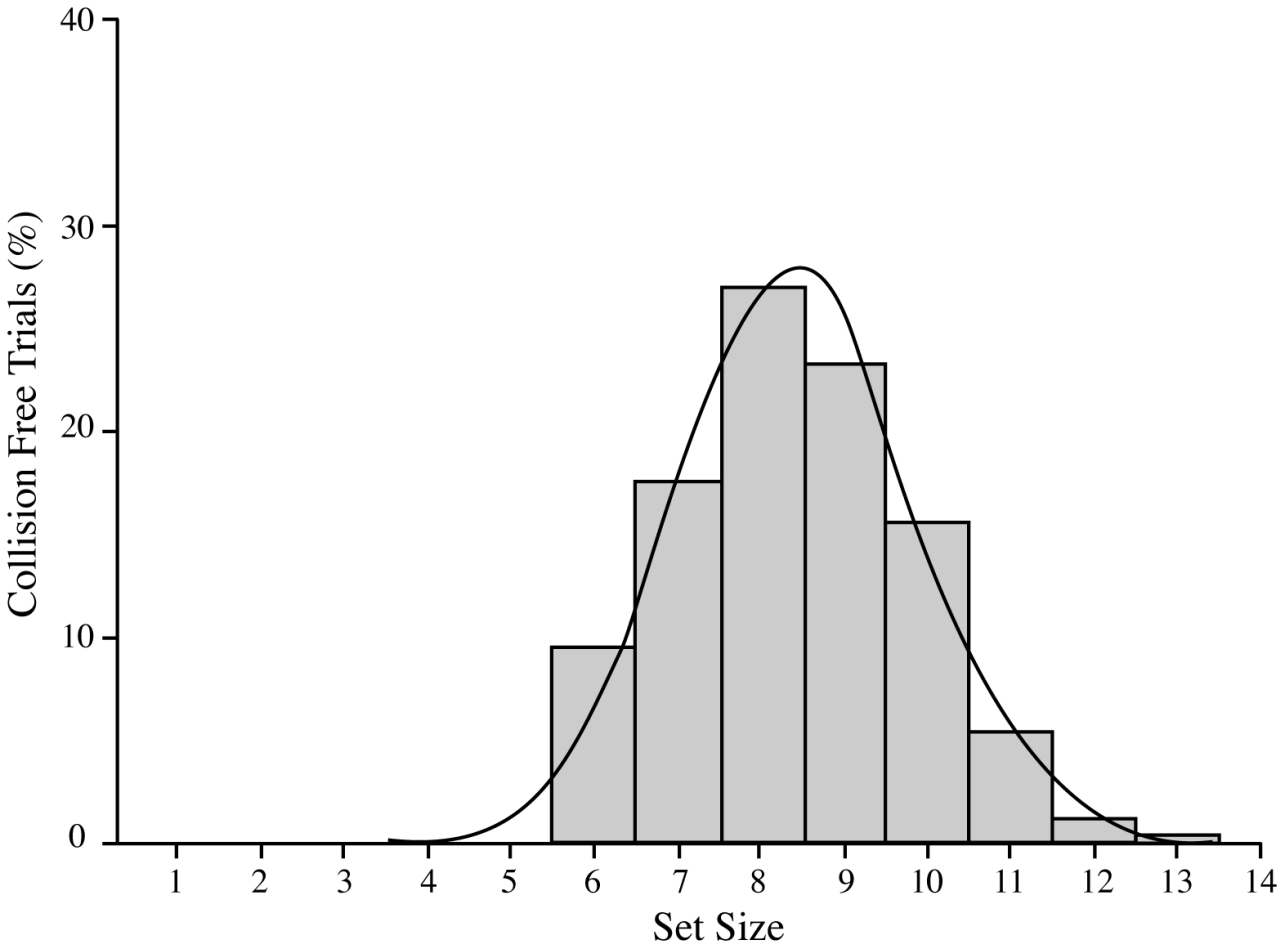
Distribution of collision-free trials in Experiment 1. Percentage of total collision-free trials, collapsed across all participants from both sites. The maximum number of items controlled was 13 and the mean number of items controlled was 8.4.

As expected, given our one-up, one-down staircase procedure, participants were collision-free on just over half of the 30 experimental trials (Mean = 16.8; SE = 0.1). Figure 3 shows the distribution of these collision-free trials as a function of set size, collapsed across all participants. It is immediately clear that the central tendency of this distribution falls at slightly above 8 items, while the maximum number of items controlled was 13. The full range of parameters extracted from the distributions of each individual participant are summarized in Tables 1 (KU participants) and 2 (SU participants).

The mean number of items that could be controlled without collision, averaged across participants, was 8.4 (SE = 0.1) and the averaged maximum value was 10.4 (SE = 0.2). These values did not vary as a function Sex or Group and there were no significant main effects or interactions.

**Figure 4:**
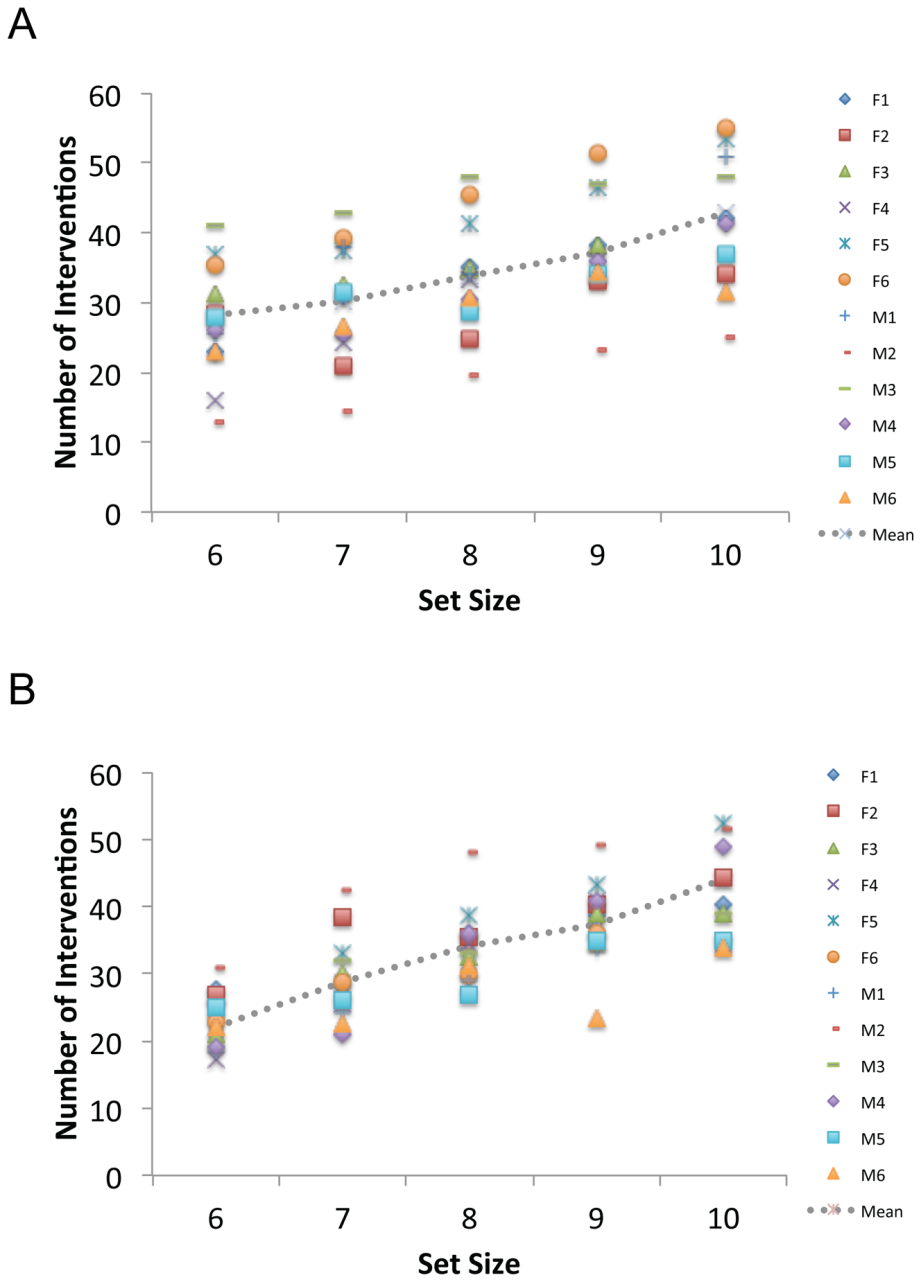
Interventions as a function of set size in Experiment 1. Data are shown separately for Korea University (KU; Panel A) and Swansea University (SU; Panel B) participants. Data are plotted for each participant using unique symbols and mean performance is represented by the dotted line. Legend codes refer to individual Female (F) and Male (M) participants in Tables 1 and 2, respectively.

Figure 4 illustrates our analyses of the number of interventions. On average, participants made just over 30 control interventions per trial (M = 34.4, SE = 1.6). However, it is clear from the distribution of symbols in Figures 4A and 4B that there were consistent individual differences in intervention strategy. For example, at the starting level of 6 items, the number of interventions across participants ranged from 13 to 41 (M = 25.1, SE = 2.0). These initial differences in intervention strategy also appear to be maintained as the number of objects in the display increases.

In general, the number of interventions increased with the number of objects participants had to control. All 24 participants had positive intervention x set size slopes, with approximately 4 additional interventions occurring each time a new item was added (M = 4.4, SE = 0.4). The mean goodness of fit for these functions was relatively high (M R^2^ = 0.8, SE = 0.1).

A 2 (Group) ×2 (Sex) ×5 (Set Size) ANOVA on the average intervention data revealed only a significant main effect of Set Size, F(4,64) = 59.6, MSE = 13.6, p<0.001, eta_2 = 0.8. Analysis of the Slope and Baseline values revealed no main effects or interactions.

To explore whether interaction style related to collision performance, we performed a multiple regression analysis with slope and baseline as predictors and mean number of items controlled as the criterion variable. Interaction behavior appeared to contribute very little to the overall success of object control, R^2^ = 0.1, F(2, 21) = 1.0, MSE = 3.2, n.s.

### Discussion

There are several findings of interest from this experiment. First, as with standard MOT, it is clear that participants were able to divide their attention between multiple dynamic objects. Here, rather than tracking the objects to identify them, participants were able to monitor the display for impending collisions and execute appropriate actions. Thus, we have shown that attention can be divided across multiple objects in both active and passive contexts.

Second, this ability to control objects and avoid collisions was clearly limited; we found that participants could only control approximately 8 items without collisions. We do not assume that this is a hard limit on human performance on this task. As with MOT [12], [15], [34], we assume that stimulus parameters such as object speed and display density will modulate levels of performance; we will address this issue in Experiment 2. Clearly, however, given any fixed parameter set, we would expect a clear upper limit on how many objects can be controlled. In the current experiment, although there was some individual variation, the estimate of 8 items was surprisingly stable. In particular, we found no variation across experimental site, suggesting that cultural differences play little role in this task. There was no reliable difference between the sexes, although as can be seen in Tables 1 and 2, there was a trend for Male participants to outperform Female participants, a theme we return to in the next experiment.

These results bring up two questions. First, what is responsible for the eight item limit? In MOT, several explanations have been proposed for the capacity limit, including a fixed set of virtual pointers [13]–[14], flexible attentional resources [15], and limitations in oscillation phase space [16]. What might underlie the limitations on iMOT performance? One hypothesis is that iMOT is relying on the same processes that subserve MOT, and therefore whatever explains MOT limitations will explain iMOT limitations. Another possibility is that the iMOT limit is purely a product of the limitations of the motor system. A third option is that the limit is a product of an interaction between the attentional and motor systems. We will return to this question in Experiment 3.

Second, what is the role of intervention strategy? The increase in interventions with set size is easy to understand, since with increasing density, the number of potential collisions is presumably increasing. We also observed consistent individual differences in the number of interventions that were maintained across variations in task difficulty. This is consistent with the suggestion raised in the introduction that some participants may adopt a more *reactive* intervention strategy and others a more *proactive* strategy. Perhaps more surprisingly, however, we found no clear relationship between intervention style and collision performance. We return to this issue in the General Discussion.

## Experiment 2

The goal of Experiment 2 was to directly compare the ability to actively control objects in iMOT with passive tracking ability as measured by MOT. A new group of Swansea students were asked to complete both tasks in separate blocks of trials. We modified the iMOT task in order to ensure that the visual characteristics of the two types of display were as similar as possible (details are given below). As in Experiment 1, a staircase procedure was used to provide individual estimates of the number of objects that could be tracked/controlled. Our main interest was in how estimates for MOT and iMOT performance would compare given identical displays. In addition to examining overall level differences, we also correlated the performance of individual participants as a first step in determining whether the two tasks appeared to draw on similar resources. We also assessed the impact of the iMOT display modifications by directly comparing performance estimates with those obtained in Experiment 1.

### Method

The equipment and viewing conditions were identical to those described in Experiment 1.

#### Participants

A group of 16 younger adults (eight female and eight male) aged between 21–32 years (M = 24.3, SD = 3.2) took part in this study on a voluntary basis. They were recruited directly from the Psychology Department at Swansea University. All participants gave written informed consent, and the methods and procedures conformed to the ethical guidelines set out by the Declaration of Helsinki for testing human participants. All aspects of the procedure was reviewed and approved by the Ethics Committee at Swansea University.

#### iMOT

We modified the iMOT stimuli and task mechanics in order to make it more compatible with the typical MOT task. We made three changes to the stimuli. First, we increased object speed from 1°/s to 2°/s. Second, we changed from a circular starting arrangement to a random distribution. Finally, we began the session with four objects, rather than six.

In Experiment 1, a trial would terminate as soon as there were any collisions, whereas in a typical MOT task, participants are asked to track for a fixed duration. In order to avoid different overall trial durations for the iMOT and MOT tasks, we changed the iMOT procedure so that each trial continued for the full trial duration, and participants were instructed to minimize the number of collisions. Concurrently, we decreased the trial duration from 30 s to 20 s. For staircase purposes, any trial with at least one collision was considered an error trial, which would result in a reduction of the set size on the subsequent trial.

We did make one significant change to the method. In Experiment 1, participants drew new paths for the stimuli, which appeared as visual traces. This would create an obvious visual difference between iMOT and MOT. We eliminated the visible paths, and simplified the control actions, such that objects could be “nudged” in any direction. Touching an object and dragging in any direction would cause the object to immediately change direction and follow the appropriate (non-visible) linear path for a random duration.

As in Experiment 1, we provided status information during each trial. At the top center of the display was a timer that provided a countdown from 20 s to 0 s. At the bottom left of the screen was the trial number, and aligned with this in the center was a text indication of whether the current session was a ‘Training’ or ‘Testing’ block of trials. At the bottom right of the display was a counter that indicated the number of collisions that occurred during the trial. Participants were instructed to minimize this number. In order to avoid visual clutter, we omitted the counter of the number of control interventions.

#### MOT

The stimuli in the MOT task were identical to those described for the iMOT in the previous section. Instead of asking the participant to prevent collisions, however, participants were asked to track a subset of the objects, the targets, during the motion phase of the trial, and then identify these targets at the end.

Each MOT session began with four objects, two targets and two distractors, randomly distributed across the display. The target objects were highlighted for three seconds by rapidly blinking at the start of the trial. For the remainder of the trial, targets and distractors were visually identical. All objects moved at 2°/s for 20 s, following the algorithm described in Experiment 1. Participants could not affect the trajectory of these objects.

At the end of the trial, the participant was asked to indicate all of the targets by touching them. If the participant correctly identified all targets, one target and one distractor would be added on the next trial. If any errors were made, one target and one distractor were subtracted on the next trial, down to a minimum set size of four items. Feedback was provided during practice trials by blinking the actual targets once a selection had been made. This feedback was not provided during the experimental blocks. Participants were instructed to try to maintain the highest possible level of performance throughout experiment.

#### Procedure

The order of the two tasks was counterbalanced between participants. We note that as block order did not affect the results in any way, this factor will not be discussed further. Before the start of each block, specific instructions were given and participants were allowed to practice until they felt comfortable with the task and response method. Each block consisted of 25 trials, and self-paced breaks were offered between trials to minimize fatigue.

### Results

#### iMOT – number of controlled items

**Figure 5:**
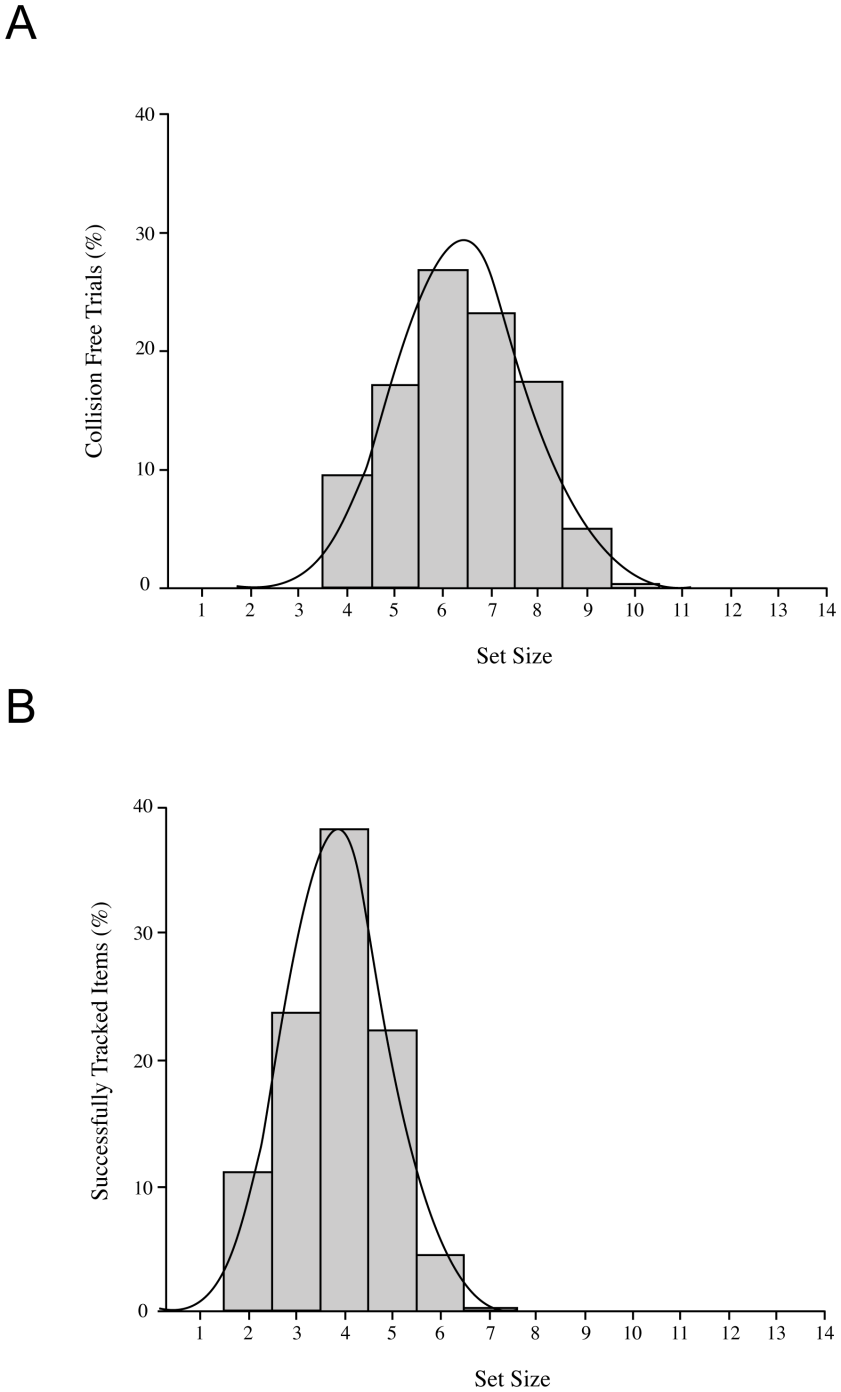
iMOT and MOT performance in Experiment 2. Panel A is the distribution of collision-free trials for the iMOT task, which has a mean of 6.4 and a maximum of 10. Panel B is the distribution of successfully-tracked trials for the MOT task, which has a mean of 3.9 and a maximum of 7.0. Data are collapsed across participants.

As before, the staircase procedure ensured that participants were collision-free on just over half of the experimental trials (Mean = 13.9; SE = 0.2). Figure 5A shows the distribution of collision-free trials as a function of set size, collapsed across participants. It is immediately clear that the overall level of performance has shifted down, relative to Experiment 1 (cf. Figure 3). The central tendency of the distribution in Figure 5A is approximately 6 items, while the maximum level achieved is 10 items.

**Figure 6:**
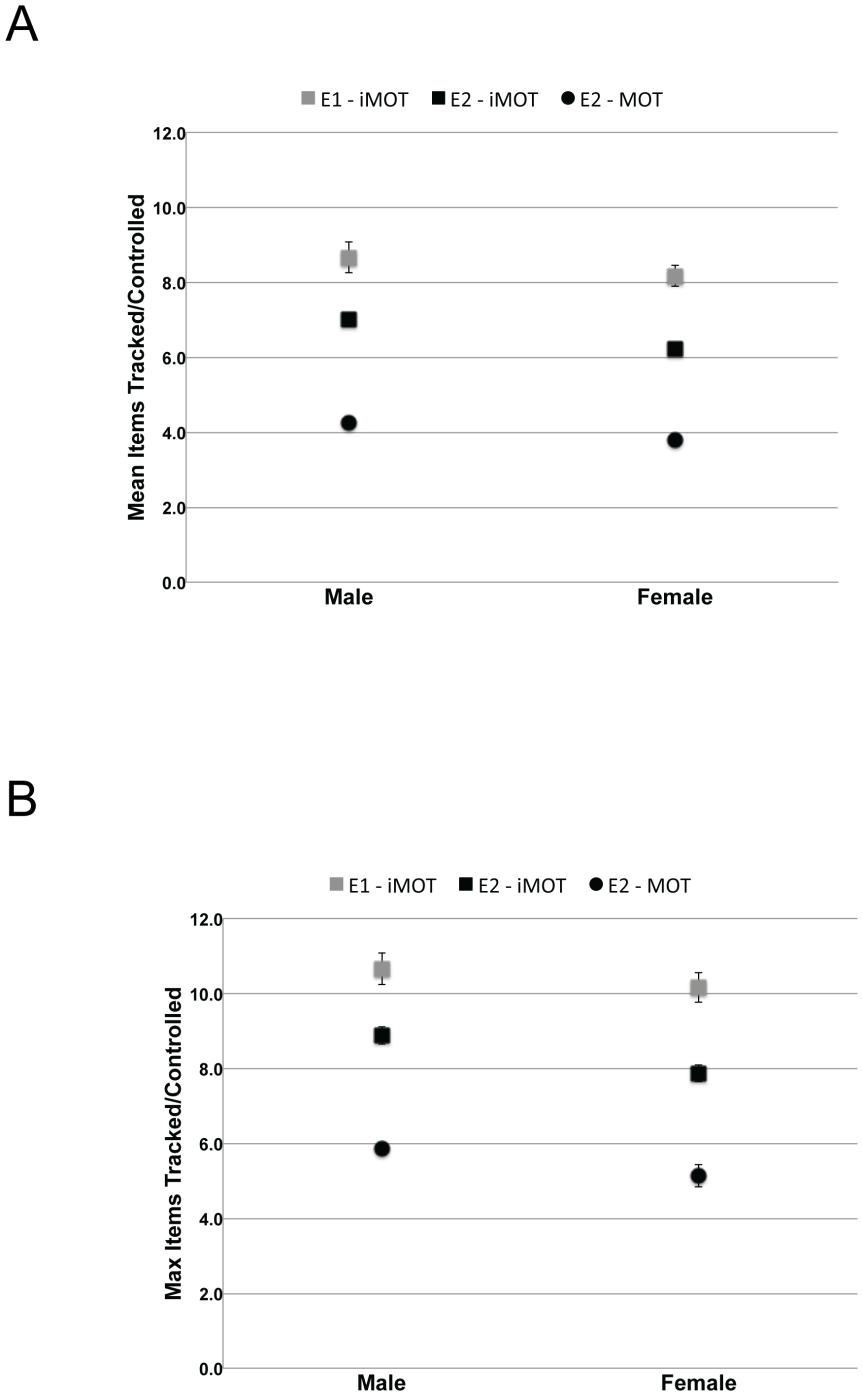
Performance in Experiments 1 and 2 as a function of participant sex. Panel A depicts mean number of tracked/controlled items and Panel B the maximum number of items tracked/controlled. Gray squares denote iMOT data from Experiment 1, black squares denote iMOT data from Experiment 2, and black circles denote MOT data from Experiment 2. Error bars denote the standard error of the mean; error bars are smaller than plotting symbols in some cases.

As in Experiment 1, we extracted a range of parameters from the distributions of individual participants (Table 3). A 2 (Experiment) ×2 (Sex) between-subjects ANOVA was used to analyze the Mean and Maximum level achieved, using the current data and those from Experiment 1 (see Figure 6). In terms of the average number of items that could be controlled, this analysis confirmed that the task used in Experiment 2 (Mean = 6.6; SE = 0.2) led to lower estimates than those obtained in Experiment 1 (Mean = 8.3; SE = 0.2), F(1,24) = 76.7, MSE = 0.3, pEta = 0.8, p<0.001. A similar pattern was found in relation to the Maximum number of items that could be controlled (Max_Exp2 = 8.4, SE = 0.2; Max_Exp1 = 10.4, SE = 0.2), F(1,24) = 28.6, MSE = 15.9, pEta = 0.6, p<0.001.

**Table 3:** *interactive* Multiple Object Tracking (iMOT): Individual Parameter Estimates for Distributions of Collision-free Trials in Experiment 2.

	Female						Male					
Part.	Count	Mean	Var	Skew	Kurt	Max	Count	Mean	Var	Skew	Kurt	Max
1	13	6.38	1.76	−0.85	−0.22	8	14	7.07	1.76	−1.07	1.13	9
2	15	6.07	1.64	0.80	0.86	9	12	7.33	1.52	−0.42	−0.45	9
3	14	5.93	0.69	−0.80	1.16	7	14	7.07	1.92	−0.96	0.43	9
4	13	6.23	1.19	−0.08	0.67	8	15	7.67	3.38	−0.86	−0.52	10
5	14	6.14	1.21	−0.32	−0.42	8	15	7.33	1.95	−1.24	1.22	9
6	14	5.86	0.75	−0.53	0.24	7	14	6.07	1.30	0.20	−0.12	8
7	14	6.57	1.65	−0.57	−0.55	8	14	6.57	1.19	−1.05	1.26	8
8	14	6.50	1.35	−0.52	0.20	8	14	6.93	1.76	−0.77	0.59	9
Mean	13.88	6.21	1.28	−0.36	0.24	7.88	14	7.01	1.85	−0.77	0.44	8.88
SE	0.24	0.09	0.15	0.20	0.23	0.24	0.35	0.19	0.26	0.17	0.28	0.24

Another feature of these data is also obvious in Figure 6. Collapsed across experiments, Male participants consistently out-performed Female participants, both in terms of mean number of items controlled (Mean_Male = 7.8, SE = 0.1; Mean_Female = 7.2, SE = 0.1), F(1,24) = 10.2, MSE = 0.3, pEta = 0.3, p<0.01 and maximum number of items controlled (Max_Male = 9.8, SE = 0.2; Max_Female = 9.2, SE = 0.2), F(1,24) = 5.9, MSE = 0.7, pEta = 0.2, p<0.05. There were no interactions between Sex and Experiment, as can be seen by comparing the individual gray (Experiment 1) and black (Experiment 2) squares in Figure 6.

#### iMOT – interventions

**Figure 7:**
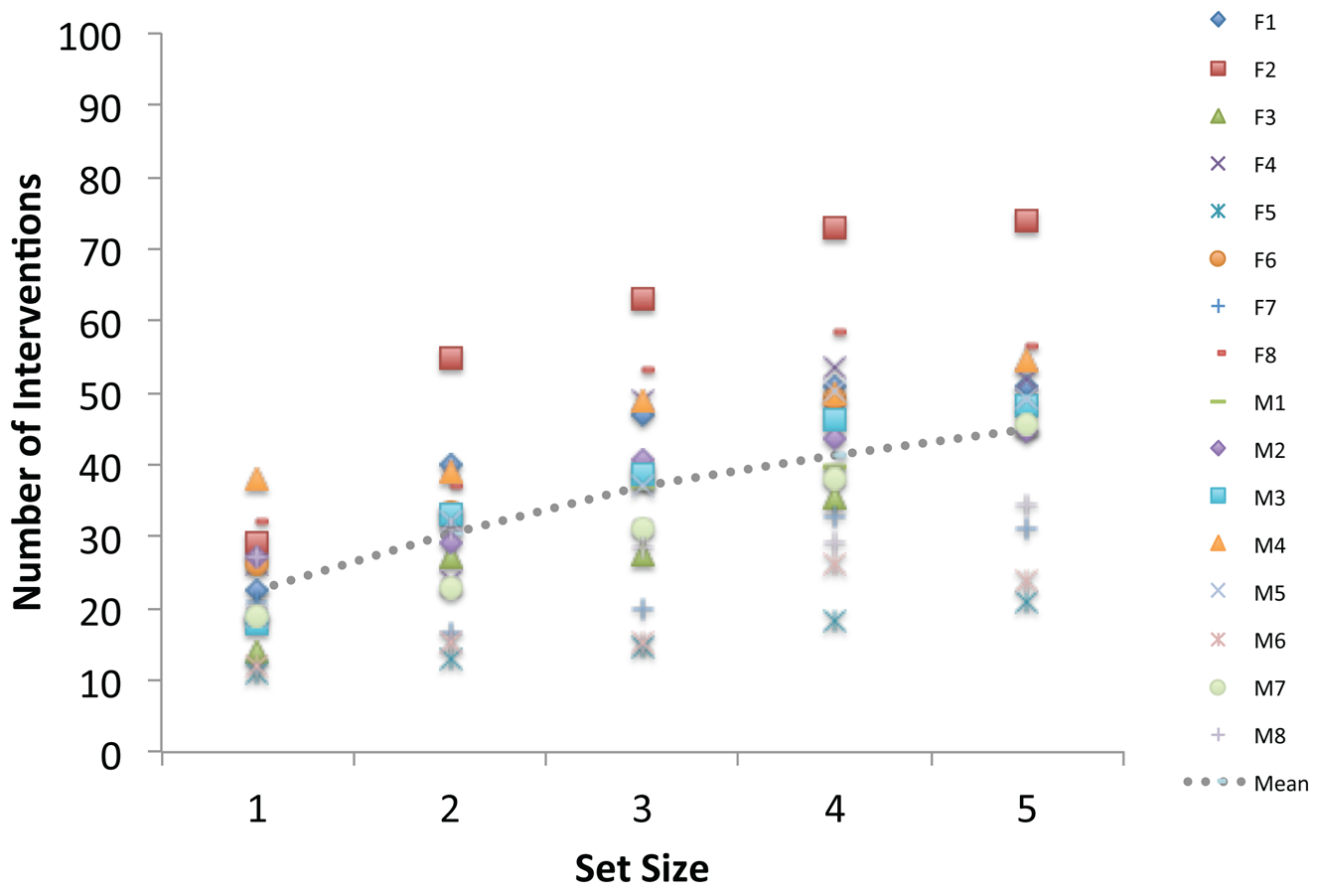
Interventions as a function of set size in Experiment 2. Data are plotted for each participant using unique symbols and mean performance is represented by the dotted line. Legend codes refer to individual Female (F) and Male (M) participants in Table 3.

Participants made close to 40 control interventions per trial (M = 38.8, SE = 3.2). As can be seen in Figure 7, there was again considerable between-participant variation in the level of interventions. At the starting level of 4 items, the number of interventions ranged from 11 to 38 (M = 22.5, SE = 1.8) and this variability appears to be maintained as set size increases. There was a relatively sharp increase in interventions as a function of set size (M Slope = 5.6; SE = 0.4) with a mean goodness of fit of 90% (M R^2^ = 0.9, SE = 0.03). As in Experiment 1, neither the slope nor the baseline interventions at level 4 had any predictive relationship with the number of items that could be controlled, R^2^ = 0.1, F(2, 13) = 1.1, MSE = 0.3, n.s.

#### MOT – number of tracked items

Figure 5B shows the distribution of successful tracking trials as a function of set size, collapsed across all participants. Participants were able to track approximately 4 items (M = 4.0, SE = 0.1) with a maximum of just over 5 (M = 5.5, SE = 0.2). Table 4 shows the full range of parameters extracted from the individual MOT distributions. As with the iMOT data, Male participants did slightly better than Female participants both in terms of Mean (Male = 4.4, SE = 0.1; Female = 3.8, SE = 0.2), t(14) = −2.6, p<0.05, and Maximum (Male = 5.9, SE = 0.1; Female = 5.1, SE = 0.3), t(14) = −2.3, p<0.05, number of items tracked. These patterns can be clearly seen in Figure 6.

**Table 4:** Multiple Object Tracking (MOT): Individual Parameter Estimates for Correctly Identified Targets in Experiment 2.

	Female						Male					
Part.	Count	Mean	Var	Skew	Kurt	Max	Count	Mean	Var	Skew	Kurt	Max
1	14	3.43	0.73	−0.18	−0.30	5	15	4.40	1.26	−0.59	0.04	6
2	13	3.23	0.53	−0.39	−0.76	4	14	4.14	1.21	−0.32	−0.42	6
3	14	4.57	1.80	−0.18	−0.09	7	14	4.14	1.05	−0.32	0.40	6
4	14	3.86	0.59	−0.91	1.86	5	14	4.07	1.30	−0.52	0.12	6
5	14	3.71	0.99	−0.42	−0.55	5	14	4.57	1.19	−1.05	1.26	6
6	14	3.71	0.53	−0.89	1.53	5	14	4.50	1.35	−0.52	0.20	6
7	13	3.77	0.69	−0.53	0.52	5	14	3.79	0.64	−0.61	0.80	5
8	14	4.14	0.75	−1.14	1.75	5	15	4.47	1.12	−1.15	0.81	6
Mean	13.75	3.80	0.83	−0.58	0.49	5.13	14.25	4.26	1.14	−0.63	0.40	5.88
SE	0.17	0.16	0.16	0.14	0.41	0.32	0.17	0.10	0.08	0.12	0.20	0.13

#### iMOT versus MOT

To examine the relationship between iMOT and MOT, we conducted two sets of analyses. First we directly compared the parameter estimates for the two tasks using a series of 2 (Task) ×2 (Sex) mixed model ANOVAs. Second, we examined whether individual performance across the two tasks was correlated.

For the Mean data, there was a significant main effect of task, with iMOT performance (Mean = 6.6; SE = 0.1) outstripping MOT performance (Mean = 4.0; SE = 0.1), F(1,14) = 53.1, MSE = 0.2, pEta = 0.9, p<0.001. There was also a main effect of Sex with Male participants (Mean = 5.6; SE = 0.1) performing slightly better than Female participants (Mean = 5.0; SE = 0.1), F(1,14) = 24.7, MSE = 0.1, pEta = 0.6, p<0.001. There were no interactions.

There were also simple main effects for the Maximum level achieved, both for Task (iMOT_Mean = 8.4; SE = 0.2; MOT_Mean = 5.5; SE = 0.2), F(1,14) = 119.5, MSE = 0.6, pEta = 0.9, p<0.001 and Sex (Male_Mean = 7.4; SE = 0.1; Female_Mean = 6.5; SE = 0.13), F(1,14) = 22.9, MSE = 0.3, pEta = 0.6, p<0.001. Again, there were no interactions.

**Figure 8:**
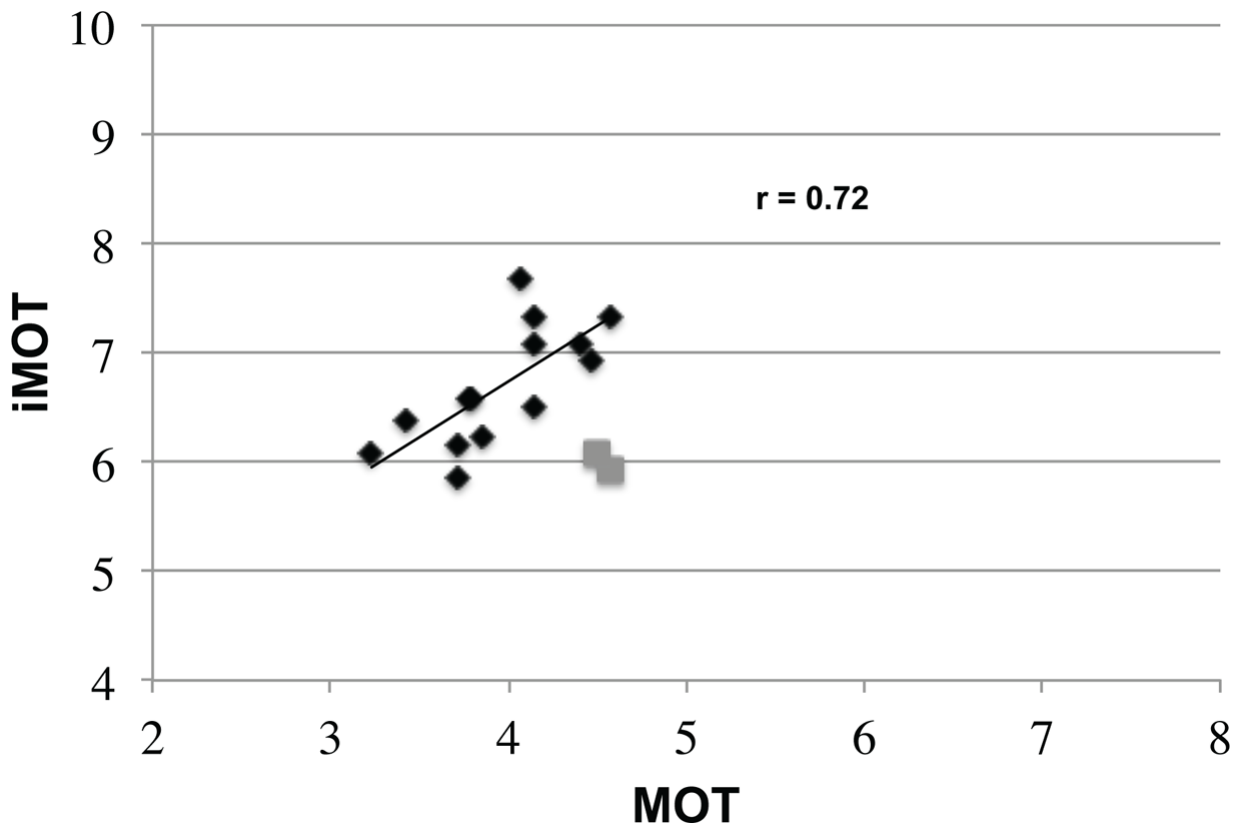
Relationship between iMOT and MOT performance in Experiment 2. MOT performance is plotted on the x-axis and iMOT performance on the y-axis. The black line denotes the regression line derived from the 14 participants represented as black diamonds. Two participants (gray squares) were omitted from the analysis (see text).

Figure 8 shows the relationship between mean iMOT & MOT performance for this group of observers. An initial analysis including all 16 observers showed a positive correlation that was not statistically significant, r(16) = 0.37, p = 0.16. Visual inspection of the data in Figure 7 shows that two participants (grey symbols) had a bimodal pattern of performance that was quite distinct from the other 14 participants. Regression analysis confirmed that these were the only two cases with standardized residual errors more than 1.5 standard deviations from the mean, suggesting that they strongly influenced the overall pattern. Excluding these data points gave rise a strong positive correlation that was significant, r(14) = 0.72, p<.01. Thus, for at least the majority of participants, those that did well on the iMOT would also be predicted to do well on the MOT task and vice versa.

### Discussion

The main aim of Experiment 2 was to compare standard MOT tracking performance with the active control performance of iMOT. Our participants were able to track approximately 4 items in the standard MOT condition, consistent with typical MOT findings [9]–[10]. The novel finding was that in the active iMOT task, with identical display characteristics, the same participants could control 6 items without collision. In both the iMOT and the MOT, there were clear sex differences, with Male participants consistently outperforming Female participants. We return to this pattern of sex differences in the General Discussion.

Note that in the iMOT condition, performance was reduced relative to Experiment 1, from approximately 8 to 6 objects. This is probably due to both the increase in speed in this experiment, and the constraint of “nudging” rather than “guiding” objects. The effect of speed is fairly intuitive: faster moving objects leave less time for the participant to react to impending collisions, and there is more uncertainty as to where to aim finger movements. Note that MOT performance is also sensitive to speed [15], [33].

One problem in directly comparing iMOT and MOT performance is the presence of distractors in the MOT task. A participant controlling four objects in iMOT is dealing with a four-object display, while a participant tracking four objects in MOT is dealing with an eight-object display. This fundamental difference between the two tasks clearly limits the conclusions that can be drawn from directly comparing absolute levels of performance. For example, additional display crowding and/or the need to allocate resources to “suppress” distractors could clearly influence estimates of MOT performance [34]–[37]. One solution would be to include both distractors and targets when estimating MOT performance. In this case one could argue that MOT performance actually outstrips iMOT performance in the current experiment. While this may prove to be a more equitable way to compare performance levels in later studies, it also clearly deviates from the way MOT capacity is usually reported.

A potentially important finding from Experiment 2 is the suggestion of a positive relationship between performance on the iMOT and MOT tasks. That is, better MOT performance appeared to predict better iMOT performance. Such a pattern is not consistent with the notion that active control and passive tracking are completely divorced. Rather, it suggests they may be drawing on similar resources. Clearly, some caution is needed in interpreting this finding as our sample size is relatively small. Furthermore, at least two out of 16 participants did not conform to the overall pattern. One motivation for implementing our task on an iPad was to plan future studies using much larger sample sizes. Having a large number of participants download and run the app on their own device would provide sufficient power to establish whether the observed relationship is stable and whether the participants we have treated as “outliers” are just that, or rather reflect consistent variability in task strategy or individual differences.

If we take these two findings at face value – partially shared resources and the ability to control more objects than can be tracked – what might this tell us about the underlying mechanisms? While we can only speculate, the most parsimonious explanation would be for the two tasks to draw on similar resources for object localization, but for the inclusion of action to bring additional precision to this localization and/or additional mechanisms that supplement overall levels of performance.

Finally, we also note that while MOT performance appeared to predict iMOT performance, the intervention strategy on the iMOT task itself did not. As in Experiment 1, there were clear individual differences in intervention strategy. The lack of a clear relationship between these strategies and collision avoidance suggests that object tracking and detection of impending collisions are more critical to success on the iMOT task than precisely how the collisions are avoided. Thus, the task would seem to more heavily on attention and perception than on motor processes. In the next experiment we continue to explore the relationship between iMOT and MOT.

## Experiment 3

The goal of Experiment 3 was to directly test the hypothesis that iMOT and MOT rely on a common mechanism by asking participants to perform both tasks simultaneously. In contrast to Experiments 1 and 2, we fixed the number of target items at four, and manipulated difficulty by changing the number of distractor items. In addition to the four target items, there could be four, eight, or twelve distractor items, randomly interleaved within a block of trials. There were three blocks in total. The first two blocks were single-task blocks in which participants performed only iMOT or MOT. In a final block of trials, both tasks were carried out at the same time, on the same stimuli. Critically, participants could only control the four target items. In the single-task iMOT condition, the target items were visually distinct from the other objects, but in the dual-task condition, they were identical to the distractors and could only be differentiated if the participant was correctly tracking. Note that this design equalizes display density between the two tasks, both under single-task and dual-task conditions.

Broadly speaking, this design can yield three outcomes. The most likely is dual-task interference, in which the dual-task condition yields performance below the single-task baseline for one or both tasks. Our strong intuition when designing this experiment was that the need to focus attention during action would disrupt the ability to successful track multiple objects. The second possibility is complete independence, in which performance in the dual-task case is equivalent to the single-task baselines. The third, rarer possibility might be termed “synergy”, in which performing the two tasks together actually improves performance on one or both tasks.

### Method

#### Participants

A new group of 16 younger adults (eight female and eight male) aged between 21–32 years (M = 24.3, SD = 3.2) took part in this study on a voluntary basis. They were recruited directly from the Psychology Department at Swansea University. All participants gave written informed consent, and the methods and procedures conformed to the ethical guidelines set out by the Declaration of Helsinki for testing human participants. All aspects of the procedure was reviewed and approved by the Ethics Committee at Swansea University.

#### Task & Design

Each participant completed three blocks of trials, single-task iMOT, single-task MOT and dual-task iMOT+MOT. On each trial there were always 4 target items. The number of distractors varied between 4, 8, and 12 items. Each block consisted of 15 trials, with five repetitions of each set-size presented in an order that was randomly determined for each participant. The order of the two single-task blocks was counterbalanced across participants and the dual-task block was always performed last. As in Experiment 2, objects moved at 2°/s, and each trial lasted for 20 s.

The single-task blocks (MOT and iMOT) were identical to the corresponding blocks in Experiment 2, with the following exceptions. First, we replaced the staircase procedure with the interleaved set-size manipulation described above. Second, during the single-task iMOT block, the four target items were drawn in blue to distinguish them from the orange distractors. Furthermore, only these target objects responded to directional control touches. We made this change in order to reduce the likelihood that participants would try to “herd” the targets and distractor items into separate areas of the screen, a strategy that could have a major impact on dual-task MOT performance. Removing touch control from distractors also helps to equate the salience of the 4 target objects across the two tasks.

The main dependent variable for the single-task MOT blocks was the number of correctly identified targets. For the single-task iMOT blocks, the main dependent variable was the number of target-distractor or target-target collisions. Distractor-distractor events did not increment the collision counter.

In the dual-task block all items were identical and were drawn in orange. The four target items were briefly flashed as in the single-task MOT trials and during the trial, only these four items would respond to touch control. Participants were instructed to try to prevent collisions between these target items and any other items. As in the single-task iMOT block, distractor-distractor collisions did not increment the collision counter. After 20 s of motion, the animation halted and participants were asked to indicate which four items were the targets by tapping them, as in the single-task MOT block. Participants were asked to perform well both tasks and we did not provide explicit instructions about which task was to be given priority.

#### Analysis

Since we did not use a staircase procedure, the dependent variables in this experiment were different than in Experiments 1 and 2. For the iMOT task, we measured the number of collisions and the number of control interventions made during the 20 s motion interval. For the MOT task, we measured the number of target items correctly identified and the overall reaction time to select the four options. Each measure was subjected to the same 2 (Sex) ×2 (Condition: single or dual task) ×3 (set-size) ANOVA. Initial examination indicated that block order did not affect performance in any way, and this factor will not be discussed further.

### Results

#### iMOT Performance

**Figure 9:**
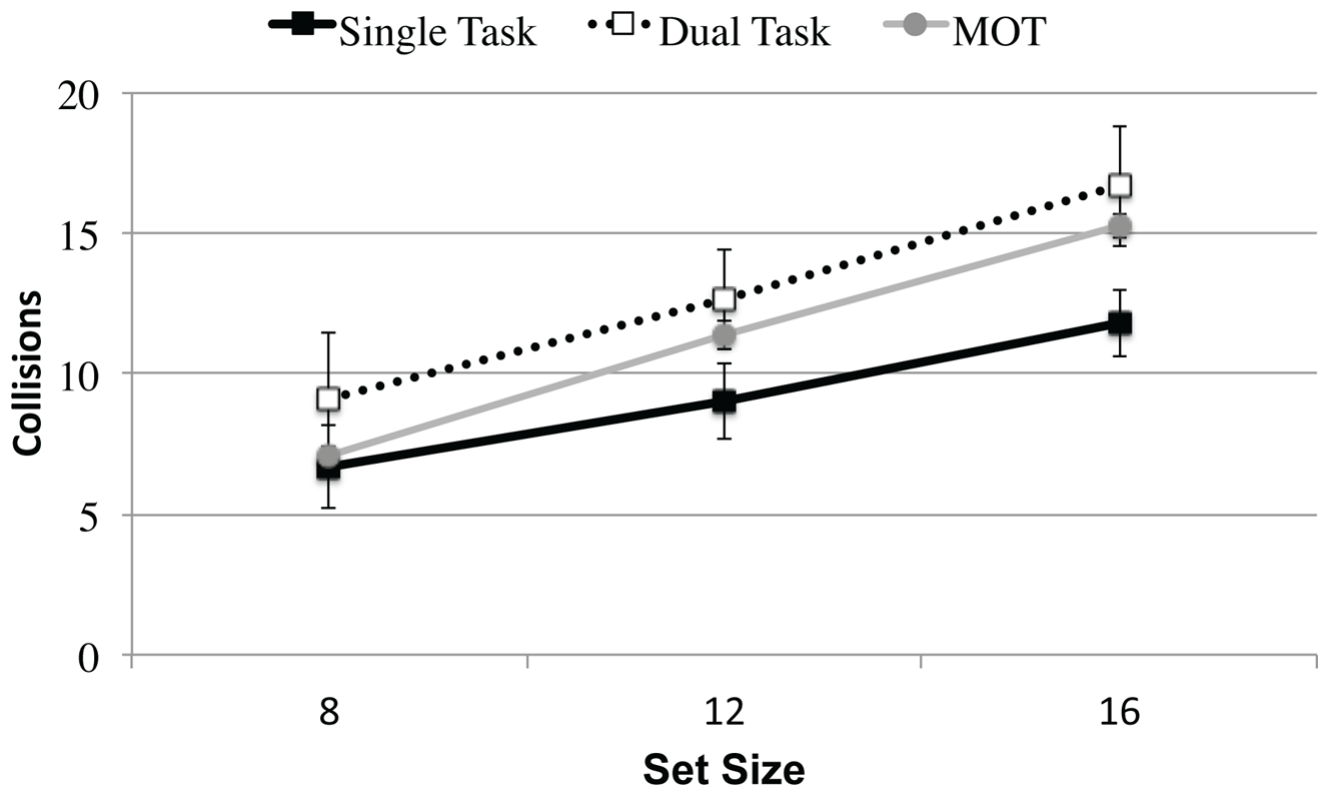
Collisions in Experiment 3 as a function of condition and set size. Dual-task performance (open squares) is clearly worse than single task performance (solid squares). Performance in the MOT condition (closed circles), in which collisions were not avoided, is plotted for comparison purposes. In all conditions, there is a clear increase in collisions as a function of set size. Error bars denote the standard error of the mean.

Figure 9 shows the number of collisions per trial as a function of set size and condition. For comparison purposes, we also plot the number of collisions from the single-task MOT block, in which participants did not interact with the targets; this provides an estimate of how many collisions would have occurred “naturally”, allowing us to determine whether participants' interventions were effective in reducing the number of collisions.

The collision data show a clear dual-task cost. There were more collisions during dual-task trials (M = 12.8, SE = 2.0) than during single-task trials (M = 9.2, SE = 1.2), F(1,14) = 8.4, MSE = 318.3, pEta = 0.4, p<0.05. The number of collisions increased with set size, with a slope of approximately 0.8 collisions/item, F(2,28) = 36.5, MSE = 321.9, pEta = 0.7, p<0.001. Although the slope appears slightly steeper for dual-task trials (M = 1.0 collisions/item, SE = 0.1) compared to single-task trials (M = 0.6 collisions/item, SE = 0.1), the Condition x Set Size interaction was not significant, F(2,28) = 1.7, MSE = 7.1, pEta = 0.1, n.s. There were no other significant effects or interactions.

Comparing the number of collisions that occurred during the passive MOT condition (as estimate of the number that would have occurred without intervention) to those that actually occurred under active conditions produces an interesting result. As can be seen in Figure 9, single-task iMOT (M = 9.2, SE = 1.2) appears to slightly reduce collisions compared to the MOT baseline (M = 11.2, SE = 0.2), reflected in marginal main effect of Condition, F(1,15) = 3.2, MSE = 103.8, pEta = 0.2, p = 0.093, and a significant Condition x Set Size interaction, F(2,30) = 3.8, MSE = 19.4, pEta = 0.2, p<0.05. In contrast, the dual-task condition (M = 12.8, SE = 1.9) actually resulted in more collisions than the MOT baseline (M = 11.2, SE = 0.2) and overall performance did not statistically differ in any way. This further illustrates the dual-task cost to iMOT performance.

**Figure 10:**
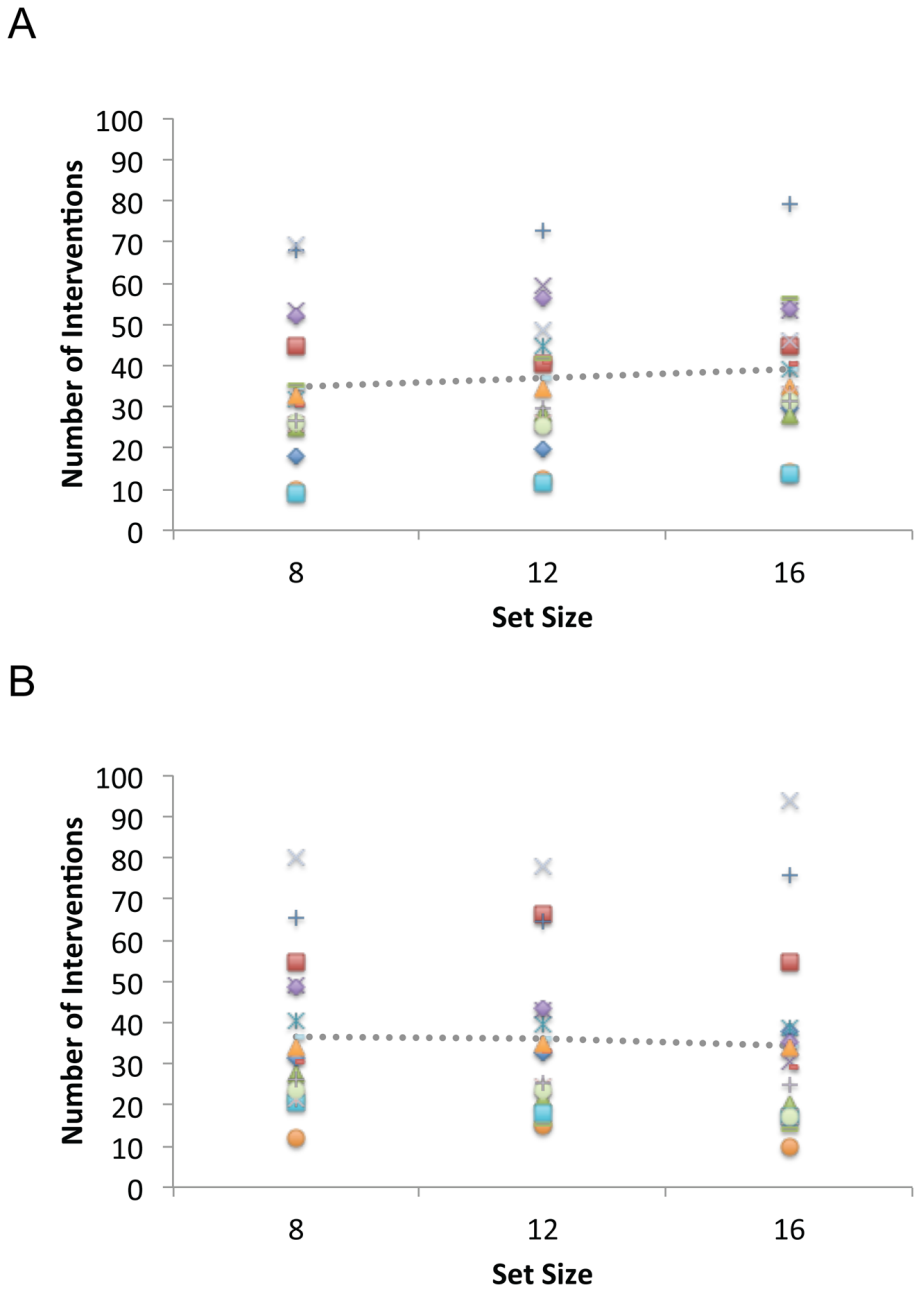
Interventions as a function of set size in Experiment 3. Data are shown separately for the single-task (A) and dual-task (B) conditions. Individual participants are represented with unique symbols and mean performance with the dotted line. There is a weak positive trend to the single-task set size data and a less coherent, negative pattern for the dual-task condition.

On average, participants made approximately 36 interventions per trial (M = 36.4, SE = 4.4). As can be seen in Figure 10, however, there was even greater between-participant variation than observed in Experiments 1 & 2. For example, in the single-task condition at the baseline set size of 8 items, interventions ranged from 9 to 69 (M = 34.8, SE = 4.7), a pattern that is maintained as the number of distractors increases. Overall, there was a slight positive slope to the single-task trials (M = 0.6, RSQ = 0.7, SE = 0.29), and a slight negative slope for the dual-task trials (M = −0.3, RSQ = 0.6, SE = 0.26). Across single- and dual-task conditions, the baseline levels of interventions were highly correlated for individual participants, r(16) = 0.9, p<.001, but the slopes were not, r(16) = −0.3, n.s.

Analysis of the average intervention data revealed a significant a Condition x Set Size interaction, F(2,28) = 3.5, MSE = 92.8, pEta = 0.2, p<0.05, confirming the presence of a dual-task change in interaction behavior. Analysis of the slope and baseline data revealed only a marginal main effect of slope, F(1,14) = 4.3, MSE = 1.3, pEta = 0.2, p = 0.057. No other main effects or interactions were significant for any of the dependent measures.

Examination of the relationship between interventions and collision data also reflects the dual-task influence on iMOT performance. In the single-task condition there was a non-significant, negative correlation between average interventions and average collisions, r(16) = −0.3, p = 0.27. In the dual-task condition, however, there was a clear positive correlation, r(16) = 0.8, p<0.001. Thus under dual-task conditions, those participants who interacted more frequently actually collided more often. Regression analysis indicated that the slope and baseline (i.e. set size 8) intervention data did not predict the slope of the collision function, either for single-task, R^2^ = 0.2, F(2, 13) = 1.9, MSE = 0.12, n.s., or dual-task R^2^ = 0.2, F(2, 13) = 1.9, MSE = 0.04, n.s., conditions.

#### MOT Performance

**Figure 11:**
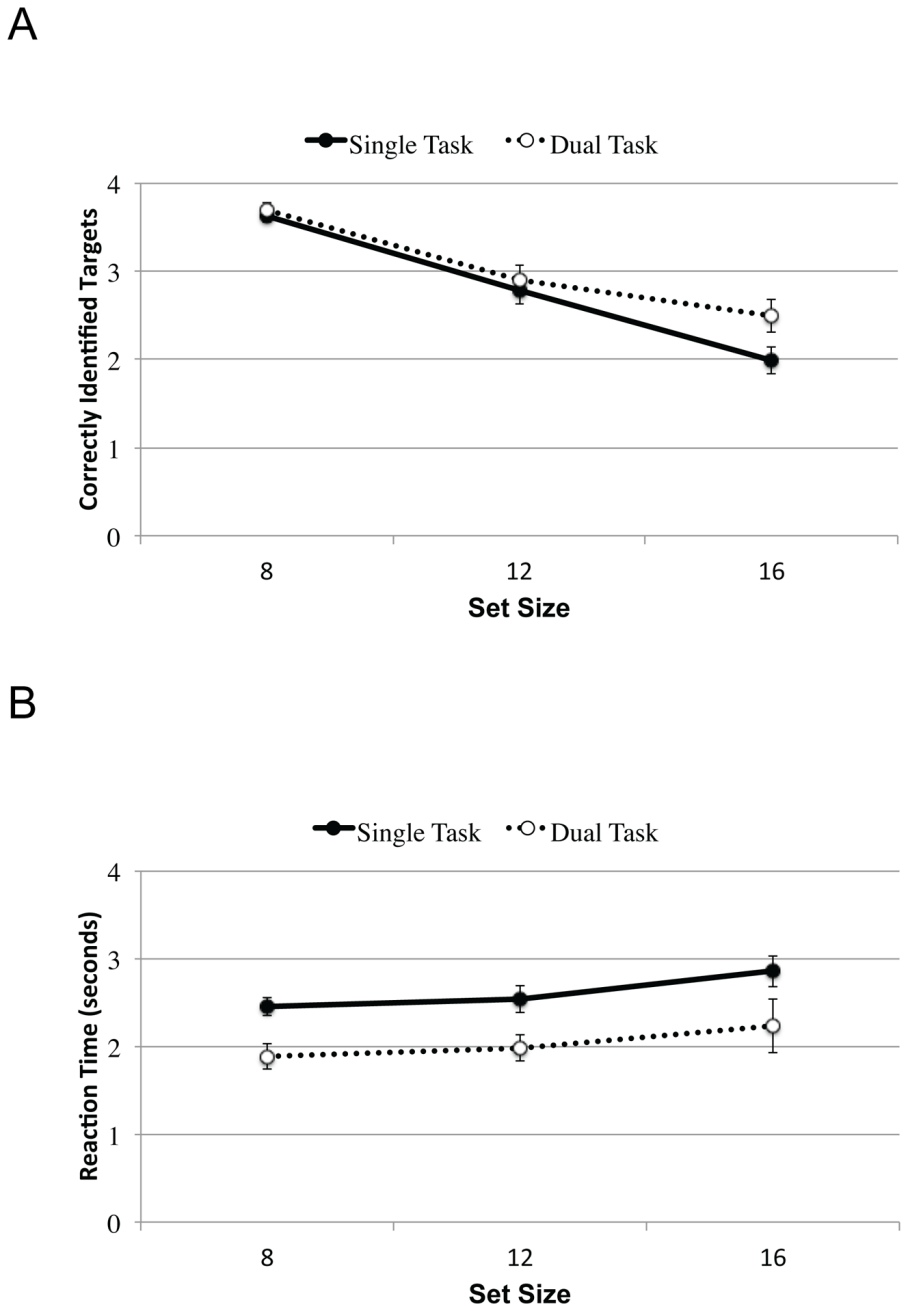
MOT performance as a function of set size in Experiment 3. Panel A shows accuracy in terms of the mean number of identified targets. Performance drops with increasing number of distractors, but less steeply under dual-task (open symbols) than single-task (closed symbols) conditions. Panel B shows reaction time to identify the targets in the final display. Across all levels of set size, dual-task responses are consistently faster than single-task responses. Error bars denote the standard error of the mean.

Figure 11 shows the data from the MOT task, with accuracy plotted as a function of set size and condition in panel A, and reaction time (RT) as a function of set size and condition in Panel B. In contrast to the iMOT results, there was a dual-task benefit for MOT accuracy. Collapsing across set size, dual-task performance (M = 3.0, SE = 0.1) exceeded single-task performance (M = 2.8, SE = 0.1), F(1,14) = 5.6, MSE = 1.3, pEta = 0.3, p<0.05. As expected, accuracy dropped as set size increased, F(2,28) = 85.8, MSE = 16.2, pEta = 0.9, p<0.001. The dual-task advantage increased as a function of set size, F(2,28) = 3.8, MSE = 0.5, pEta = 0.2, p<0.05, probably due to ceiling effects at set size 8. The only other effect to reach significance was a main effect of Sex, with Male participants (M = 3.1, SE = 0.1) correctly identifying more targets than Female participants (M = 2.7, SE = 0.1), F(1,14) = 5.0, MSE = 3.9, pEta = 0.3, p<0.05.

A similar dual-task benefit is observed in the RT data, where dual task responses (M = 2.0 s, SE = 0.2) were consistently faster than single task responses (M = 2.6 s, SE = 0.1), F(1,14) = 13.1, MSE = 8.1, pEta = 0.5, p<0.01. RTs also increased as a function of Set Size, F(2,28) = 5.4, MSE = 1.2, pEta = 0.3, p<0.05, with a slope of approximately 47 ms/item. No other effects or interactions were significant.

### Discussion

Consistent with the results of Experiment 2, the current findings suggest that iMOT and MOT rely, at least to some extent, on common underlying mechanisms. Specifically, dual-task performance was clearly modulated relative to the singe-task baseline conditions. The precise pattern of this modulation, however, is both surprising and intriguing. That is, we observed a dual-task *cost* for iMOT, and a dual-task *benefit* for MOT. Explaining this asymmetrical finding and what it might more generally tell us about the impact of action when attention is divided will be a main focus of the General Discussion, which we return to shortly.

Several other features of the data from Experiment 3 are also worth commenting on. For example, single-task iMOT performance at first glance seems worse than would be expected from Experiments 1 & 2. Given that participants in Experiment 2, on average, could control 6 items with no collisions on half of the trials, why were participants in Experiment 3 encountering roughly 6 collisions per trial while only controlling 4 items? The answer probably has to do with the fact that only the 4 target items could be controlled, in Experiment 3, whereas participants could control all of the items in Experiment 2. As mentioned above, we made this change to avoid “herding” behavior, but it clearly may also have had an impact on overall collision avoidance. Alternatively, the drop in performance may have arisen due simply to the target/distractor distinction that was not present for Experiments 1 and 2. Although collision with any item had to be avoided, the existence of two “sets” of objects may have invoked additional processes, such as distractor suppression, that could have influenced iMOT performance over and above dual-task costs *per se*.

As in Experiments 1 and 2, there was considerable variability in the level of interventions made between participants. Indeed, here the spread of interventions seemed even greater, and was still obvious even under dual-task conditions, when control behavior seemed to break down. This latter finding is at least suggestive that the tendency to touch the device might relate more to idiosyncratic motor preferences, rather than to collision avoidance strategies.

The clear sex differences seen in Experiment 2 were only present in the single-task MOT condition of the current experiment. In contrast, in both single and dual-task iMOT condition, Female participants recorded less collisions, although these differences were not significant.

Finally, we should note that this experiment is only a first step in exploring the dual-task relationship between iMOT and MOT. Additional studies will be needed in order to more fully explore this relationship while controlling for additional factors. Explicitly manipulating the priority of one or other task, by requiring zero iMOT collisions or 100% MOT tracking performance, for example, would be a useful approach for testing the limits of shared resources. Similarly, returning touch control to all objects, would remove the ability to recover MOT targets by hand. As we discuss in more detail below, we believe the current dual-task study has already shed light on the ability to act while dividing attention, but clearly further studies will need to confirm and extend our results.

## General Discussion

In this paper we have introduced a new task, interactive Multiple Object Tracking (iMOT), designed to measure the ability to actively track and control a set of identical targets. In Experiment 1 we showed that participants were able to divide their attention between multiple targets over extended periods of time and, in addition, were able to plan and execute actions designed to control objects in order to avoid collisions. As with MOT, this ability appears to be limited, with the precise limit varying depending on display conditions such as speed (Experiment 1 versus 2) and object density (Experiments 1–2 versus 3).

In Experiment 2, we showed that when the same group of participants perform iMOT and MOT under identical display conditions, they can actively control more items than they can passively track. This experiment also demonstrated that for 14 out of 16 participants, there was a positive correlation between the two tasks, suggesting possible common underlying mechanisms.

In Experiment 3, using a dual-task design, we found converging evidence for a relationship between the two tasks. However, rather than a simple pattern of dual-task interference, we found a dual-task *cost* for iMOT, and a dual-task *benefit* for MOT. As mentioned in the introduction to Experiment 3, finding “synergy”, where performance on a task actually improves under dual-task conditions, is quite rare.

### Dual task benefits for MOT

How might we explain the dual-task benefit for MOT observed in Experiment 3? Previous research has demonstrated that object collisions and/or close approaches between items are a major source of errors in MOT [12],[35]–[37]. As the goal of iMOT is to avoid collisions, the presence of interventions could indirectly improve MOT performance by increasing object spacing. The only problem with this explanation is that collisions actually increased in the dual-task condition relative to those that occurred by chance in the single task MOT condition. This makes object separation seem an unlikely explanation for the dual-task benefit.

Another possibility is that action is being used as an additional tagging mechanism to improve localization and identification. Pylyshyn's seminal account of MOT [13] proposed that the ability to track multiple objects simultaneously reflected the existence of virtual mental pointers or indexes, used for deictic reference in spatial computations (see also [37]). Pylyshyn coined the term FINSTs to refer to these pointers, from “FINgers of INSTantiation”. Perhaps there are additional “fingers” brought into play to represent the action targets of our physical fingers during dual-task trials?

A more prosaic possibility is that the dual-task condition allows participants to test hypotheses about which items are targets and which are not. Imagine that you lose track of one of the targets. In the single-task MOT condition, there is no way, except by chance, to recover from this loss. However, in the dual-task condition, you can touch an item, and if it responds, then it's a target, if it doesn't respond, it's a distractor. We restricted touch control in this way in order to reduce the possibility that participants would strategically segregate the display, pushing all distractors to one area of the screen and all targets to another. As an aside, we should note that it remains possible that targets alone were “herded” in this way in order to make them more easily available for tracking.

In any event, it remains a possibility that being able to identify targets by touch accounts for some or even all of the dual-task benefit we observed in Experiment 3. Certainly, this additional avenue for recovering targets could be obscuring more general dual-task costs for the MOT task. Of course, in order for this factor to fully account for the dual-task MOT data, we would have to assume that all of our participants ignored instructions to track and simply used touch to probe for possible targets. Had this been a common strategy we might have expected performance to remain fairly constant across set size, which was not the case. Similarly, none of our participants reported that they stopped tracking during the dual-task trials, although we did not ask specific debrief questions. Clearly, in future studies it would be useful to attempt to control “herding” by other means, such as more variable trajectories, and to reinstate touch to all objects.

Although we have been focusing on MOT accuracy, RT data in Experiment 3 also showed a consistent dual-task improvement. Participants were approximately 600 ms faster to locate the targets in the dual-task than the single-task condition, suggestive of improved confidence in localization. As the dual-task block always followed the single-task block, it is possible that some of this increase in speed is simply familiarity with the method of responding. Similarly, with the current design, we cannot rule out the possibility that some component of the dual-task benefit is simply a MOT-specific practice effect.

Whatever the cause, it is clear that task-relevant action does not appear to greatly disrupt MOT performance as measured in Experiment 3. However, as already mentioned, we need to be cautious in generalizing from these findings as the “target-recovery” possibility afforded by touch could be artificially inflating estimates of MOT dual-task performance. We thus feel it remains a real possibility that in other settings the need to focus attention in order to act could prove disruptive for tracking multiple objects in parallel [44], an outcome we alluded to in the introduction to Experiment 3. The contribution of the current work is that it makes a first attempt to examine whether actions can be planned and executed at the same time as tracking multiple objects. Further studies will be required to establish whether the apparent enhancement of tacking generalizes to situations where additional cues to target identify are more tightly controlled. We return to more generally implications for action and divided attention below.

### Dual task costs for iMOT

Next, we turn to the dual-task cost observed for iMOT. Compared to single-task conditions, the need to separate target and distractor sets – both to complete the standard MOT task and to control objects – could have decreased the resources available for collision avoidance. Similarly, as previous research has suggested that successful MOT performance involves distractor suppression [34]–, this could have had consequences for action. That is, if suppression operated by inhibiting the spatial location occupied by a distractor, then when target and distractor approach one another this may reduce the effectiveness of actions. For example, it may still be possible to “touch” a target, but not to plan effective avoidance manoeuvers in an inhibited part of space. This could explain both the increase in collisions and the reduction in interventions as a function of set size, since more of the collisions would be of a target-distractor nature as the distractor∶target ratio increased.

Finally, we cannot rule out the possibility that participants simply strategically allocated resources to MOT at the expense of iMOT. Although we instructed participants to perform equally well in both tasks, the need to explicitly identify the four targets in the final phase of each trial and/or the clear demands of maintaining tracking-for-identity may have shifted priority on to MOT. Also, we should note that our fixed target set size of 4 items would have been close to capacity in terms of MOT, but below capacity in terms of iMOT, based on estimates from Experiment 2. This may also have resulted in priority being given to the MOT task. To test these ideas, it might be possible to shift priority to iMOT by terminating trials after the first collision, as in Experiment 1, or by manipulating the target set size so that MOT is less demanding. If the current pattern of dual-task costs and benefits reflects strategic allocation, we might expect iMOT to show “synergy”, and MOT interference, under such conditions.

**Figure 12:**
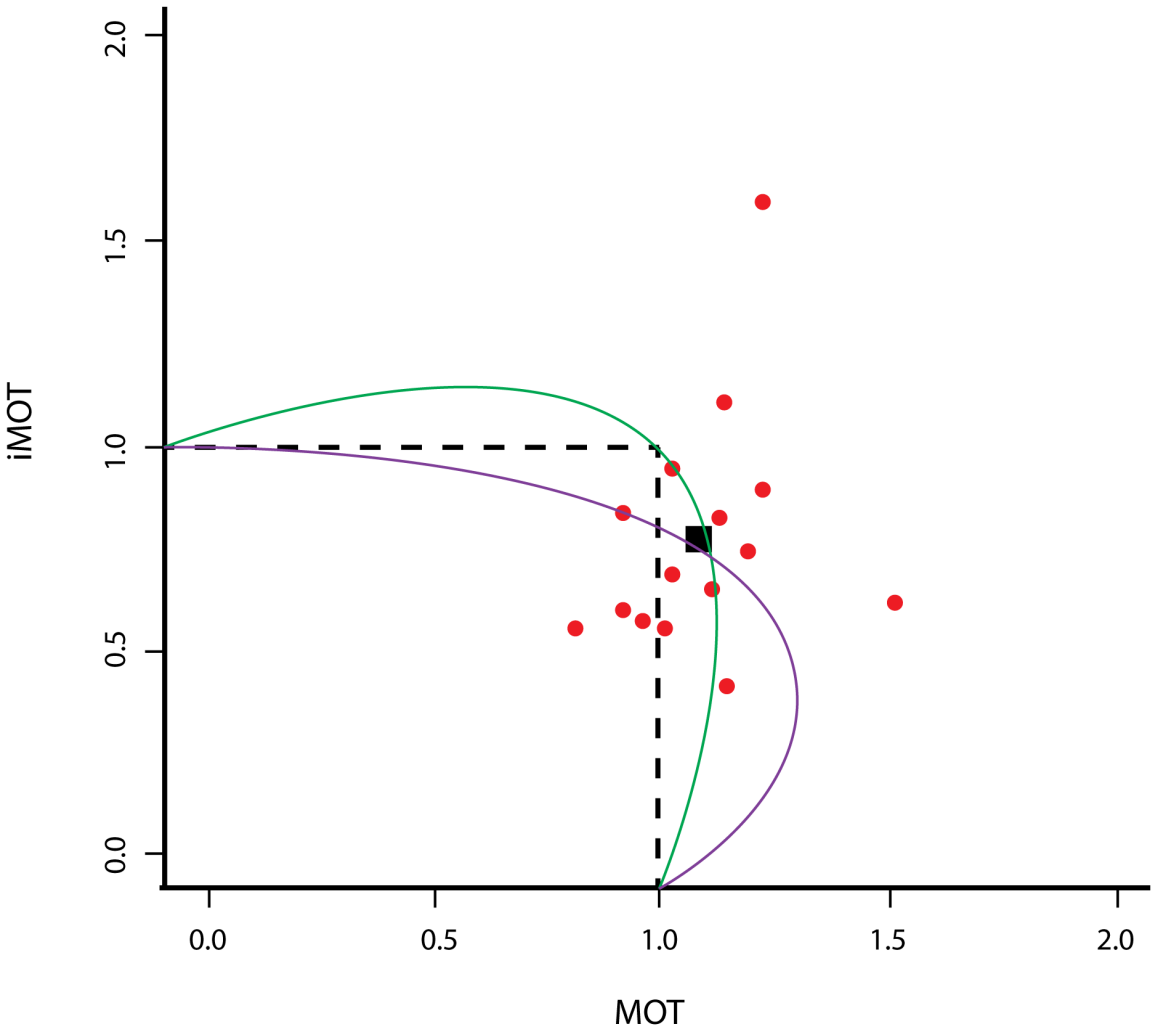
Attentional Operating Characteristic for Experiment 3 data. The dashed line shows the typical AOC space, so that the vertical line indicates MOT single task performance, and the horizontal line iMOT single task performance. Data falling at the intersection indicates independence, data inside the box indicates dual-task costs and data outside of the box indicates “synergy”. The red points indicate dual-task performance for each individual participant. Data have been transformed relative to single task performance. MOT performance is thus dual-task targets correct as a proportion of single-task proportion correct. iMOT data have been similarly transformed, except inverted, as higher collision scores ordinarily represent worse performance. Equivalent dual and single task performance on both measures would thus result in a score of 1.0. The black square is the overall mean. The purple curve represents the family of AOC curves in which concurrent MOT always hurts iMOT, but there is a range over which MOT gets a benefit. The green curve is a symmetrical solution, where, depending on how participants allocate priority between the two tasks, we could observe synergy on both tasks, or costs for MOT and benefits for iMOT.

On a relate point, it is important to keep in mind that so far we have only measured one point along the attentional operating characteristic (AOC) between iMOT and MOT. Generally speaking there are two basic families of AOC curves that could be compatible with the data from Experiment 3. These are shown in Figure 12. The first family of possible AOC curves would be inherently asymmetrical, such that dual-task iMOT is always at a disadvantage relative to single-task iMOT, while dual-task MOT may be advantaged or disadvantaged, depending on the priority given to iMOT (purple curve in Figure 12). In the second alternative (green curve in Figure 12), the characteristic is essentially symmetrical, and either iMOT, MOT, or both could benefit from synergy, depending on priority allocation.

The current data does not allow us to definitively exclude either possibility. Average performance indicates that there exists a region in the AOC space in which iMOT performance is degraded, while MOT performance is improved. However, the presence of at least two participants with better dual-task performance in *both* MOT and iMOT, and the better overall fit to our data would tend to favor a symmetrical solution. Clearly, future studies will be needed to more fully explore this AOC space.

### Integrating attention and action

An important goal of this paper was to explore the influence of action when attention is distributed rather than focused. As mentioned in the Introduction, previous studies have shown that planning or executing an action has clear consequences in the context of focused attention [21]–[22]. Similarly, attention is thought to play an important role in some aspects of motor learning and control [45]–[46]. Our interest in the current paper was in whether the need to act in order to control specific targets would conflict with the need to distribute attention across the whole display in order to track and monitor for impending collisions.

The findings of Experiments 1 and 2 indicate that these two components can be integrated in our new task to support performance that is equal to or even exceeds those observed in passive tracking alone, suggesting little conflict. Single-task iMOT performance, then, would appear to demonstrate that action has little impact on standard estimates of the ability to divide attention. We should note, however, that in these initial studies, it remains possible that participants were serially deploying attention to individual targets [47] rather than distributing attention in parallel across the whole display [44].

Indeed, in Experiment 3, when concurrent MOT demands required parallel tracking, both the quantity and the quality of control actions were reduced. A goal of future studies will be to determine whether this apparent conflict reflects fundamental differences in tracking per se (i.e., serial versus parallel tracking) or reflects demands placed by additional processes, such as distractor inhibition or fine motor control.

Clearly, one of the main novel features of iMOT is the need to control objects. What have the current studies told us about this active component of performance? First, in all three experiments there was considerable between-participant variation in intervention strategy. Participants seemed to adopt a particular level of intervention and to maintain this level regardless of subsequent increase in difficulty. That is, differences in baseline levels of intervention appeared to outweigh the set size slopes in all of the experiments. This behavior might reflect quite stable preferences to be *reactive* or *proactive* in dealing with impending collisions. An interesting avenue for future studies will be to attempt relate such strategic behavior to more general individual differences [48].

Second, individual intervention strategy did not a appear to be a good predictor of task success. Indeed, the only dataset when there appeared to be a clear relationship was in the dual-task condition of Experiment 3. Here, more interventions were associated with more collisions. In general, it would appear that iMOT can support a range of intervention strategies and further research will be needed to establish how the tracking and prediction demands of the task relate to motor planning and execution. In addition to strategies relating to collision avoidance, it may be that participants are trading off number of interventions for some other quantity that we did not think to measure, for example the spatial or temporal precision of motor movements.

Remaining with the topic of individual differences, previous research has indicated that male participants might outperform female participants on MOT tasks [35]. MOT accuracy data from Experiments 2 & 3 confirm this finding. The iMOT data in Experiments 1 & 2 also showed a similar pattern, with male participants outperforming female participants. In Experiment 3, however, although there were no reliable differences, in both the single- and dual-task condition, female participants recorded fewer collisions than male participants. In future studies it will be interesting to establish whether these patterns reflect the use of different strategies, as suggested by [43] in which case they might easily be overcome with training [40], or whether they reflect more fundamental differences in spatial cognition [41].

### A few notes on the iPad as an experimental platform

The current work represents our group's first attempt to conduct research using a mobile device, such as an iPad. There are a number of features that lead us to believe such devices will become commonplace in laboratories that need to measure human performance. Here, we list a few observations that might prove useful for others' contemplating experimental work in this area. As the devices are relatively cheap and available, it is possible to equip a lab with a number of identical experimental devices, even given a quite limited budget. As “mobile” devices, they enable extreme flexibility in where studies are carried out, making it possible to make the most of limited lab space and to go offsite to work with special populations (e.g., in clinics, homes, schools). Of course, control over environmental conditions (e.g., lighting, extraneous noise) can become problematic. The same is true if applications are designed to be downloaded on to personal devices, rather than lab devices. The appeal here, of course, is the potential to collect data from very large samples of the general public.

In general, it is relatively easy to design and implement experiments via device-specific development environments (e.g., XCode) or third party, device-independent software such as Unity 3D. As the devices themselves are built to display high quality video and to have response times that can support real-time game-play, they would appear suitable for many types of experiments. At least on some devices, obtaining accurate technical benchmarks can be problematic, when precise control of display or response are needed. However, we expect that both the specifications of such devices and access to technical material will continue to improve.

Our impression is that participants approached the current tasks with a very different attitude to standard screen-and-keyboard tasks. Clearly, we designed these experiments to be game-like. Even beyond this, holding and controlling the device seemed to change the dynamics of conducting the study in a way that made the participants seem more engaged and at-ease, a feature that may important for populations beyond typical young adults.

Finally, we have began to explore the potential of indirect measures of behavior made available through built in hardware, such as the iPad's 3-axis accelerometer. We noted above that direct measures, such as the number of interventions, were poor predictors of overall performance. In pilot testing for the current work, we used the accelerometer to record individual differences in device orientation and the force applied during each touch. Unsurprisingly, increases in task difficulty led to more interventions and greater overall force being applied. Unexpectedly, we found reliable correlations between device tilt and collision avoidance, with flatter orientations leading to better performance [49]. We believe that such indirect assessments of performance could have great potential as additional measures of the mind.

## Conclusions

How does action affect performance when tracking multiple objects? We found that active tracking (iMOT) actually increased participant's functional capacity, relative to passive tracking (MOT). Active and passive tracking do seem to share processing resources, insofar as participants who were better at one also tended to be better at the other. Finally, the two processes interacted in an intriguing fashion under our experimental conditions: while actively avoiding collisions improved MOT performance, explicitly keeping track of target locations impaired the ability to control objects. These findings suggest that our ability to track multiple objects is not just a clever attentional trick for playing perceptual shell games. Instead, these same processes can be harnessed to effectively manipulate multiple objects in the world. Conversely, the inherently serial nature of action does not appear to constrain our parallel attentional processes. Attention and action cooperate to allow us to interact with our dynamic environment.
